# Epidemiology, treatment, costs, and long-term outcomes of patients with fireworks-related injuries (ROCKET); a multicenter prospective observational case series

**DOI:** 10.1371/journal.pone.0230382

**Published:** 2020-03-19

**Authors:** Daan T. Van Yperen, Cornelis H. Van der Vlies, J. Tjeerd H. N. De Faber, Xander Smit, Suzanne Polinder, Charlotte J. M. Penders, Esther M. M. Van Lieshout, Michael H. J. Verhofstad, ROCKET study group

**Affiliations:** 1 Trauma Research Unit Department of Surgery, Erasmus MC, University Medical Center Rotterdam, Rotterdam, the Netherlands; 2 Burn Center, Maasstad Hospital, Rotterdam, the Netherlands; 3 The Rotterdam Eye Hospital, Rotterdam, the Netherlands; 4 Department of Plastic, Reconstructive and Hand Surgery, Erasmus MC, University Medical Center Rotterdam, Rotterdam, the Netherlands; 5 Department of Public Health, Erasmus MC, University Medical Center Rotterdam, Rotterdam, the Netherlands; Medical University of Graz, AUSTRIA

## Abstract

**Objective:**

People in the Netherlands are legally allowed to celebrate New Year’s Eve with consumer fireworks. The aim of this study was to provide detailed information about the patient and injury characteristics, medical and societal costs, and clinical and functional outcome in patients with injuries resulting from this tradition.

**Methods:**

A multicenter, prospective, observational case series performed in the Southwest Netherlands trauma region, which reflects 15% of the country and includes a level I trauma center, a specialized burn center, a specialized eye hospital, and 13 general hospitals. All patients with any injury caused by consumer fireworks, treated at a Dutch hospital between December 1, 2017 and January 31, 2018, were eligible for inclusion. Exclusion criteria were unknown contact information or insufficient understanding of Dutch or English language. The primary outcome measure was injury characteristics. Secondary outcome measures included treatment, direct medical and indirect societal costs, and clinical and functional outcome until one year after trauma.

**Results:**

54 out of 63 eligible patients agreed to participate in this study. The majority were males (N = 50; 93%), 50% were children below 16 years of age, and 46% were bystanders. Injuries were mainly located to the upper extremity or eyes, and were mostly burns (N = 38; 48%) of partial thickness (N = 32; 84%). Fifteen (28%) patients were admitted and 11 (20%) patients needed surgical treatment. The mean total costs per patient were €6,320 (95% CI €3,400 to €9,245). The most important cost category was hospital admission. Only few patients reported complaints in patient-reported quality of life and functional outcome after 12 months follow-up.

**Conclusion:**

This study found that young males are most vulnerable for fireworks injuries and that most injuries consist of burns, located to the arm and hand, and eye injuries. On the long-term only few patients experienced reduced quality of life and functional limitations.

## Introduction

Around 1950 it became a Dutch tradition to use consumer fireworks when celebrating New Year’s Eve. Nowadays, Dutch people spend approximately 70 million euros annually on consumer fireworks [1]. Although the joyful character the use of fireworks is not without danger. Every year many patients require hospital treatment, in particular males with hand and eye injury. Nevertheless, fireworks is widely used by both adults and children [2–5].

Fireworks has the potential to cause permanent physical disability. In a series of 143 patients with fireworks-related eye injuries 15 patients went blind and 55 eyes sustained permanent damage [6]. In some cases this results in a whole body impairment up to 77% [7]. This does not only have effect on an individual’s quality of life, it also results into increased health care costs and costs due to the absence of work or lost productivity. On average each year 1–2 persons die from this tradition [2].

In the past years the public and political debate in the Netherlands about a nationwide ban on the use of consumer fireworks during New Year’s Eve has intensified. However, no reliable data about the long-term economic, clinical, and functional outcome are available.

The primary aim of this prospective case series was to report detailed information about the injuries caused by consumer fireworks. The secondary aims were to provide information about treatment, medical and societal costs, and long-term clinical and functional outcome after one year. The outcome of this study can contribute to the debate about whether or not to ban consumer fireworks.

## Methods

### Study design & setting

This multicenter, prospective, observational case series recruited patients from every hospital in the Southwest Netherland trauma region. All 13 hospitals in the area participated, including a level I trauma center, a specialized burn center, a specialized eye hospital and 10 general hospitals. Patients were included between December 1, 2017 and January 31, 2018. Although in the Netherlands it is only legally permitted to lit fireworks as a consumer during New Year’s Eve, a wider inclusion period was chosen to not miss any patient.

Each participant, or parents, provided written informed consent. Participants remained encoded during the study. This study has been exempted by the Medical Research Ethics Committee Erasmus MC (Rotterdam, the Netherlands; registration number MEC-2017-1066). The study is registered at the Netherlands Trial Register (trial registration numbers NTR6793 and NL6608; date of registration 31-Oct-2017; www.trialregister.nl).

The baseline characteristics and injury details of the participants in this study have been published elsewhere [8].

### Participants

All patients (no age limit) with any type of injury caused by fireworks treated in a hospital in the Trauma Region Southwest Netherlands were eligible for inclusion. Treatment was defined as any intervention for which at least one clinical follow-up visit was scheduled. Patients (or parents of pediatric patients) had to provide written informed consent.

Patients were excluded from participation if they had unknown contact information or insufficient understanding of Dutch or English language to understand the study documents.

After enrolment by a clinician in the local hospital, patient details were handed to the coordination investigator for further data collection and follow-up. Follow-up was performed at three, six, and 12 months after trauma. At these follow-up moments data were extracted from patient’s medical records and patients or proxy completed questionnaires. On indication patients also underwent physical examination. Data extraction and physical examination was performed by a trained investigator (DTVY).

**Fig 1:**
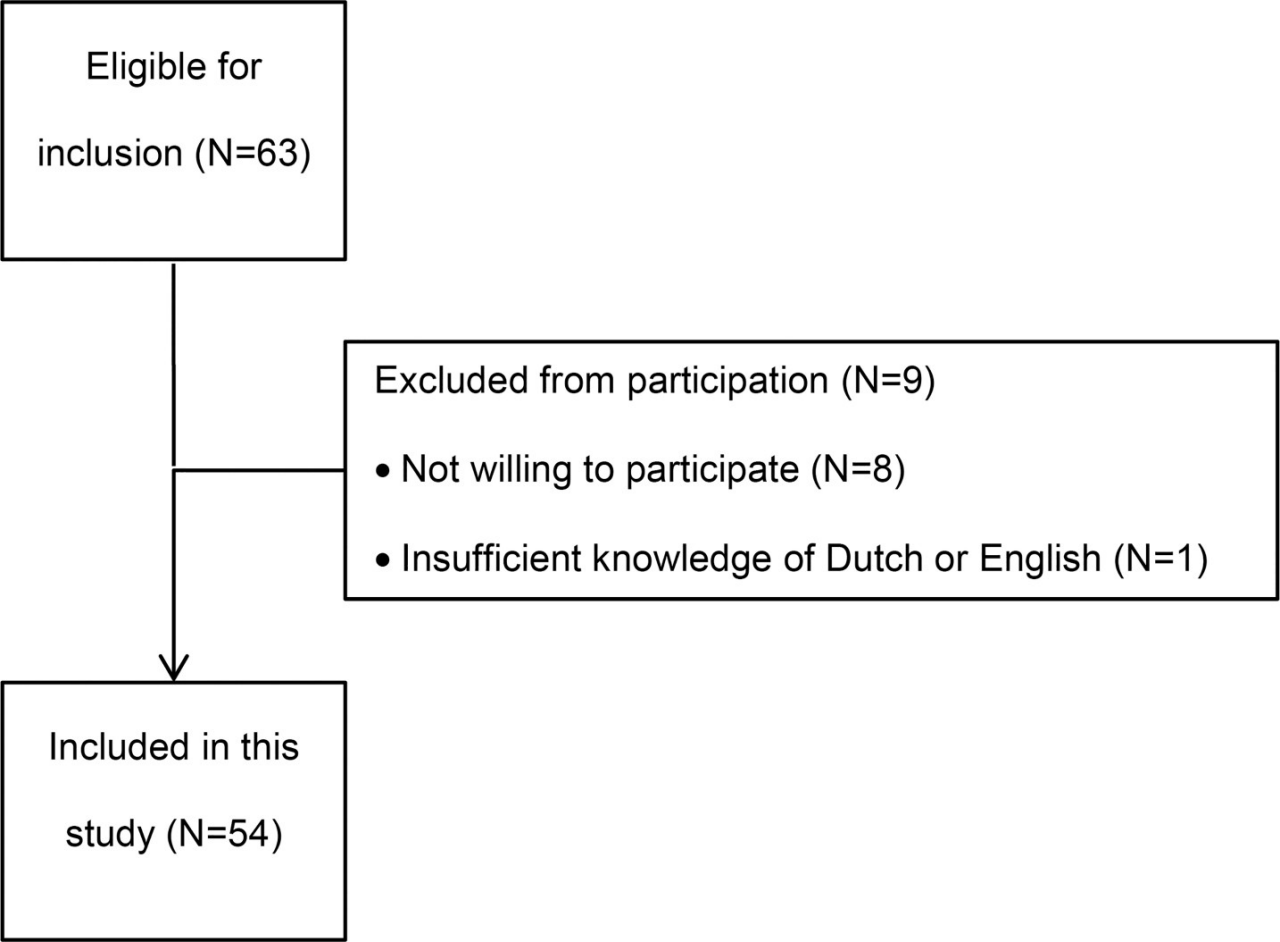
Eligibility chart.

A total of 63 patients with fireworks-related injuries were eligible for inclusion. Eight patients refused to participate and one did not understand Dutch or English, so 54 patients were included in this study (Fig 1). Median age of the study population was 15 (P_25_-P_75_ 11–25) years and half of all patients (N = 27) were children younger than 16 years (Table 1). The vast majority were males (93%), both in the pediatric and the adult subgroups: 26 (96%) and 24 (89%), respectively. Approximately half of the patients stated they were bystander (N = 25; 46%) and in 75% (N = 37) the injuries were caused by legal fireworks (N = 37; 76%). Further patient characteristics and trauma mechanism are described in Table 1.

**Table 1:** Patient characteristics and trauma mechanism.

	N*	All (N = 54)	N*	Children (N = 27)	N*	Adults (N = 27)
**Patient characteristics**						
Age (years)	54	15 (11–25)		11 (8–12)		25 (18–32)
Male		50 (93%)		26 (96%)		24 (89%)
ASA score 1		49 (91%)		26 (96%)		23 (85%)
**Accident information**						
Lit within permitted period		27 (50%)		11 (41%)		16 (59%)
Bystanders		25 (46%)		11 (41%)		14 (52%)
Legal fireworks	49	37 (76%)	26	21 (81%)	23	16 (70%)
Correctly used	43	23 (54%)	24	10 (42%)	19	13 (68%)
Type of firework	51		27		24	
Bangers		22 (41%)		14 (52%)		8 (30%)
Decorative		28 (52%)		13 (48%)		15 (55%)
Rockets		7 (25%)		3 (23%)		4 (27%)
Cakes		7 (25%)		1 (8%)		6 (40%)
Other		14 (50%)		9 (69%)		5 (33%)
Calcium carbide		1 (2%)		0 (0%)		1 (4%)

Data are shown as median (P_25_-P_75_) or as N (%).

* This represents the number of patients from whom data were available.

ASA, American Society for Anesthesiologists.

### Data collection

The primary outcome measure was injury characteristics. The number of injuries including location and type (*e*.*g*., burns, soft tissue damage, eye injury) was recorded. The secondary outcome measures were patient characteristics, accident information, treatment details, direct medical and indirect societal costs, and the patient-reported outcomes.

Patient characteristics included age, gender, medical history, and the American Society for Anesthesiologists (ASA) classification.

For the trauma mechanism the role of the patient (igniter or bystander) was noted, when the injury was sustained (within or outside the legally permitted period), the legality of the fireworks, whether the fireworks were used correctly (as instructed in the manual and without modifying the fireworks), and the kind of fireworks used (bangers, decorative fireworks, or other). A bystander was defined as a person who got injured from a piece of fireworks he did not ignite himself. All fireworks for sale at official selling points in the Netherlands were considered as legal consumer fireworks. For example sparkles, cakeboxes, rockets and calcium carbide. Age limitations apply for the sale of fireworks and the minimum age for the purchase of lightest category is 12 years [9].

Regarding the treatment characteristics the number and type of surgical interventions, and the duration of hospital length of stay including Intensive Care Unit (ICU) admission were recorded.

Patient’s electronical medical records and a customized questionnaire based on two questionnaires (Medical Consumption Questionnaire and Productivity Cost Questionnaire) validated by the institute for Medical Technology Assessment (iMTA) were used to measure the direct medical and indirect societal costs [10, 11]. Direct medical costs included all medical care directly associated with treatment and rehabilitation (*e*.*g*., hospital admission, operations, outpatient clinic, and physical therapy). Indirect societal costs included costs due to the absence from paid work. Costs for lost productivity were calculated using a friction cost method with a friction period of 85 days.

During follow-up the number of days with absence from work and school were reported. The economic evaluations were done from a societal perspective. Direct medical and indirect societal costs from work absence until one year after trauma were measured in accordance with economic guidelines [12]. Health care costs were calculated by multiplying the volumes of healthcare use with the corresponding unit prices (see S1 and S2 Tables). Cost prices were determined by bottom-up micro-costing method and all unit prices were indexed with the national consumer price index to 2018.

Patients were asked about their daily activities (sports, hobbies, and leisure activities) before trauma. During follow-up they were asked to what extend they were able to perform their activities compared with pre-trauma. The activities were noted and their ability was scored on a Numeric Rating Scale (NRS), ranging from 0 (unable) to 10 (comparable with pre-trauma level).

At three, six, and 12 months follow-up, several questionnaires were completed to measure patient-reported outcomes. For health-related quality of life the EuroQol-5D-3L (EQ-5D) was used. This is a validated tool to measure health-related quality of life, consisting of 5 items (dimensions mobility, self-care, usual activities, pain/discomfort, and anxiety/depression) and a visual analog score (EQ-5D-VAS) for self-related health status [13]. The EQ-5D was completed for all patients aged four years or older. Parents of pediatric patients aged 4 to 7 years old completed the ‘youth proxy’ version, and pediatric patients between 8 and 15 years old completed the ‘youth’ version themselves with help of their parents if necessary. The outcomes of the five dimensions and the EQ-5D-VAS were presented. The EQ-5D-VAS score ranges from 0–100 and a higher score indicates a better health status.

Patients completed additional questionnaires for specific injuries. Patients with eye injuries (adults and children) completed the Health Utilities Index Mark 3 (HUI-3), domain vision (consisting of two questions, each with four possible answers), to measure generic health status regarding their eye injury [14]. Also the Snellen visual acuity was noted, if recorded in their medical records. A score of ≥0.8 was considered a fully functional eye, a score between 0.2 and 0.8 was considered as impaired vision, and a score between 0 and 0.1 was considered a (legally) blind eye [6].

Adults and children with upper extremity injuries completed the 1-item HUI-3 dexterity question, and adults completed the 15-item *Quick* Disabilities of the Arm, Shoulder and Hand (*Quick*-DASH) which is validated to measure physical function and symptoms in patients with any or multiple musculoskeletal disorders in the upper limb [14, 15]. Sum scores were transformed to a final score ranging from 0 to 100, with a higher score indicating greater disability.

For adult patients with lower limb injuries the Lower Extremity Functional Scale (LEFS) was scored to assess a patient’s ability to perform everyday tasks (20 items with a maximum score of 80 points) [16]. A lower score indicates greater disability. For children no validated questionnaire regarding lower limb function was available.

In patients with burns (adults and children) the Patients and Observer Scar Assessment Scale (POSAS) was used to measure the scar quality. This tool consists of a patients-reported part, completed by the patient and/or parent, and an observer part, completed by a trained observer (DTVY) [17]. Both parts consist of 7 items (6 domains and 1 overall score), scored 0–10, and a lower score indicates better scar quality.

Patient’s satisfaction level regarding function and cosmesis of the injured body part was determined in adults only using an 11-point numeric rating scale, ranging from 0 (extremely dissatisfied) to 10 (fully satisfied).

Patients view on the use of consumer fireworks before and after trauma, and how the trauma influenced their opinion, was measured with a custom-made questionnaire at six months follow-up in adults only.

### Sample size calculation

A formal sample size calculation for this observational study was not made. In 2017–2018, 434 patients needed fireworks-related treatment in a Dutch hospital [2]. The Trauma Region Southwest Netherlands covers approximately 2.5 million inhabitants, and with a total population of 17 million in the Netherlands, approximately 60–70 patients were expected to be eligible for inclusion.

### Data analyses

Statistical analyses were performed using the Statistical Package for the Social Sciences (SPSS) version 25.0 (SPSS, Chicago, Ill., USA). Data are reported following the ‘Strengthening the Reporting of Observational studies in Epidemiology’ (STROBE) guidelines.

Normality of continuous data was tested with the Shapiro-Wilk test. This showed they all deviated from the standard normal distribution. A p-value <0.05 was taken as a threshold for statistical significance in all statistical tests and all tests were two-sided. Missing values were not replaced by imputation.

Descriptive analyses were performed to report the outcome measures. Data are shown for the entire population as well as for adults and children (<16 years). For continuous data, median and quartiles were reported. For categorical data, number and frequencies were reported. The costs were reported as a mean with 95% confidence interval (95% CI).

## Results

### Injury characteristics

**Fig 2:**
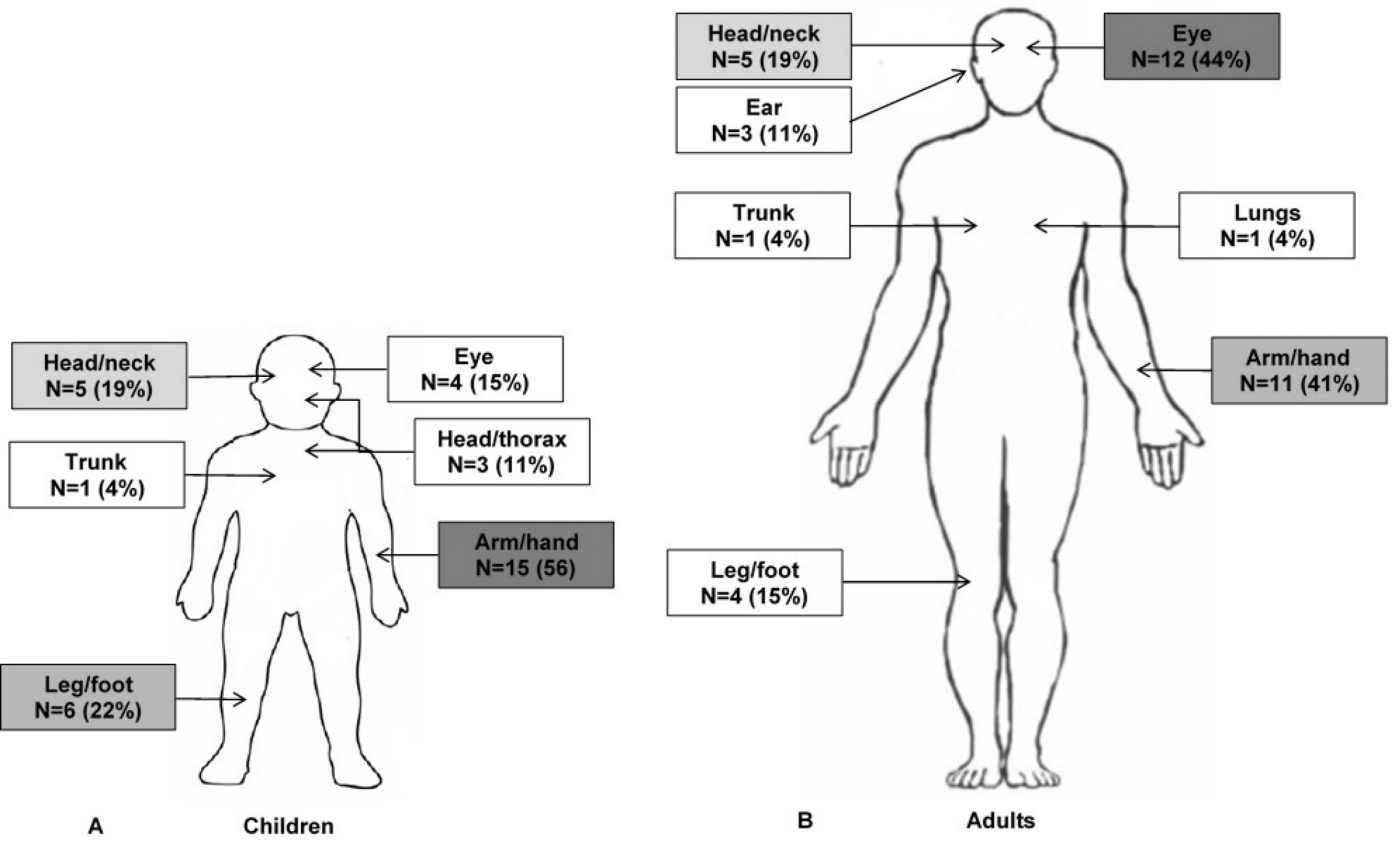
Body region involved in children (A) and adults (B) with fireworks-related injuries.

In the entire cohort a total of 79 individual injuries were reported, with a maximum of 4 per patient. The most affected body site group was the arm/hand region (N = 26), followed by the eyes (N = 16), the head/neck (N = 10) and the leg/foot (N = 10). A distinction for children and adults was made in Fig 2.

Burns were the most common injuries (N = 38; 48%), followed by eye injuries (N = 19; 24%). 84% (N = 32) of the burns were of partial thickness and 16% (N = 6) of full thickness.

Thirteen patients had one injured eye and three had bilateral eye injury. Causes were blunt (N = 14), chemical (N = 5), penetrating (N = 1), or thermal (N = 1) trauma and resulted into damage to the ocular surface (N = 13), anterior chamber (N = 9), posterior segment (N = 7) or adnexa oculi (N = 4). None of these patients wore any safety glasses.

Other injuries were soft tissue lacerations (N = 11; 14%); superficial (N = 7) and deep (N = 4), ear injuries (N = 3; 4%); ear drum perforation (N = 2) and labyrinth damage (N = 1), fractures (9%); proximal humeral shaft (N = 2), orbital roof (N = 1), distal radius (N = 1), proximal phalanx (N = 1), femoral shaft (N = 1), and tibia/fibula (N = 1), and inhalation injury (N = 1), due to the inhalation of smoke caused by fireworks. None of the patients died of their injuries.

### Treatment

Fifteen (27%) patients were admitted to a hospital. Six children were admitted for a median of nine (P_25_-P_75_ 2–15) days and nine (33%) adults for a median of five (P_25_-P_75_ 4–8) days. Two adults were admitted to the ICU for two and three days, respectively.

Eleven (20%) patients underwent operative treatment, with up to three operations per patient. Most often performed procedure was eye surgery (N = 5; 45%), followed by wound debridement (N = 4; 36%), and soft tissue reconstruction (N = 4; 36%; Table 2).

**Table 2:** Operative treatment needed for fireworks-related injuries.

	N*	All (N = 54)	N*	Children (N = 27)	N*	Adults (N = 27)
Operative treatment		11 (20%)		5 (19%)		6 (22%)
>1 surgery		7 (64%)		2 (40%)		5 (83%)
Type of surgery	11		5		6	
Debridement		4 (36%)		2 (40%)		2 (33%)
Amputation		1 (9%)		1 (20%)		0 (0%)
DIP digit 3		1 (100%)		1 (100%)		0 (0%)
Fracture treatment		3 (27%)		2 (40%)		1 (16%)
ORIF distal radius		1 (33%)		0 (0%)		1 (100%)
Open reposition digit 1		1 (33%)		1 (50%)		0 (0%)
CRIF femoral shaft		1 (33%)		1 (50%)		0 (0%)
ORIF proximal humeral shaft		1 (9%)		1 (50%)		0 (0%)
Soft tissue reconstruction		4 (36%)		2 (40%)		2 (33%)
SSG		3 (75%)		2 (100%)		1 (50%)
Eyelid correction		1 (25%)		0 (0%)		1 (50%)
Eye surgery		5 (45%)		1 (20%)		4 (67%)
Vitrectomy		4 (80%)		0 (0%)		4 (100%)
Corneal transplantation		1 (20%)		0 (0%)		1 (25%)
Foreign body removal		1 (20%)		0 (0%)		1 (25%)
Iris reconstruction		2 (40%)		0 (0%)		2 (50%)
Amnion repair		1 (20%)		1 (100%)		0 (0%)
Cataract surgery		3 (60%)		0 (0%)		3 (75%)
Baerveldt implant surgery		1 (20%)		0 (0%)		1 (25%)
Dressing change under anesthesia		1 (9%)		1 (20%)		0 (0%)

Data are shown as N (%).

* This represents the number of patients from whom data were available.

CRIF, Closed Reduction Internal Fixation; DIP, Distal interphalangeal; ORIF, Open Reduction Internal Fixation; SSG, Split Skin Graft.

### Costs and absence from work and school

The mean costs per patient per cost category are presented in Table 3. The mean total costs per patient for the entire group were €6,320. For children and adults, the mean total costs per patient were €4,210 and €8,430, respectively. Intramural costs accounted for 79% of the total costs, of which costs for hospital stay were the most dominant cost category (52%).

**Table 3:** Mean costs per patient for patients with fireworks-related injuries.

Costs category	Total costs (in €)	Costs for children (in €)	Costs for adults (in €)
Medical costs			
Intramural			
Transportation, referral and ED visit	890 (530 to 1,255)	1,125 (410 to 1,840)	660 (475 to 840)
Hospital stay (incl. ICU)	2,575 (490 to 4,660)	1,540 (0 to 3,185)	3,610 (0 to 7,560)
Surgery	710 (180 to 1,240)	440 (0 to 875)	985 (0 to 1,970)
Outpatient clinic visits	810 (615 to 1,000)	960 (650 to 1,270)	655 (420 to 890)
Subtotal	4,980 (2,465 to 7,510)	4,065 (1,360 to 6,765)	5,910 (1,475 to 10,345)
Extramural	60 (25 to 100)	80 (15 to 145)	40 (20 to 60)
**Subtotal**	**5,045 (2,515 to 7,575)**	**4,145 (1,405 to 6,880)**	**5,950 (1,515 to 10,380)**
Societal costs; work absence	1,275 (240 to 2,310)	65 (0 to 175)	2,480 (455 to 4,510)
**Total costs**	**6,320 (3,400 to 9,245)**	**4,210 (1,470 to 6,945)**	**8,430 (3,175 to 13,690)**

Costs are presented as a mean costs per patient with 95% confidence interval between brackets.

Two children and 21 adults had a paid job before their injury, and during follow-up two children and 18 adults had been absent from work, with a median of 10 and 12 days, respectively (Table 4). At final follow-up all patients returned to work. The mean costs per patient due to work absence was €1,275, and €65 and €2,480 for children and adults, respectively.

**Table 4:** Effects of fireworks-related injuries on work participation and school attendance.

	N*	All (N = 54)	N*	Children (N = 27)	N*	Adults (N = 27)
Work						
Work participation pre-trauma (N patients)	49	23 (47%)	23	2 (8%)	26	21 (91%)
Work participation pre-trauma (hours/week)	23	40 (8–45)	2	4 (3–4)	21	40 (15–45)
Work participation pre-trauma (days/week)	23	5 (2–5)	2	1 (1–1)	21	5 (3–5)
Work absence during follow-up (N patients)	27	20 (74%)	5	2 (40%)	22	18 (82%)
Work absence during follow-up (days)	20	12 (4–20)	2	11 (3-.)	18	12 (4–20)
School activities (pre-trauma)						
School attendance pre-trauma (N patients)	50	34 (68%)	24	24 (100%)	26	10 (38%)
School attendance pre-trauma (hours/week)	34	30 (25–33)	24	30 (28–34)	10	29 (8–31)
School attendance pre-trauma (days/week)	34	5 (5–5)	24	5 (5–5)	10	5 (1–5)
School absence during follow-up (N patients)	37	26 (70%)	26	19 (73%)	11	7 (64%)
School absence during follow-up (days)	26	5 (3–20)	19	4 (1–18)	7	5 (4–55)

Data are shown as median (P_25_-P_75_) or as N (%).

* This represents the number of patients from whom data were available.

During follow-up 19 (73%) children and seven (64%) adults were absent from school as a result of their injuries, with a median number of days absent of four and five, respectively.

### Return to daily activities

Before trauma, a total of 43 patients (23 children and 20 adults) reported to have any activities of daily living, with a median of 8 hours per week. From 3 months onwards, all were fully able to perform their activities on their pre-trauma level (Table 5).

**Table 5:** Changes over time in ability to perform activities of daily living on pre-trauma level, for children and adults with fireworks-related injuries.

	N*	All (N = 54)	N*	Children (N = 27)	N*	Adults (N = 27)
3 months	43	10.0 (8.5–10.0)	23	10.0 (8.8–10.0)	20	10.0 (7.8–10.0)
6 months	43	10.0 (10.0–10.0)	23	10.0 (10.0–10.0)	20	10.0 (8.2–10.0)
12 months	43	10.0 (10.0–10.0)	23	10.0 (10.0–10.0)	20	10.0 (9.5–10.0)

Data are shown as median (P_25_-P_75_).

* This represents the number of patients for whom data were available.

### Patient-reported outcome

**Fig 3:**
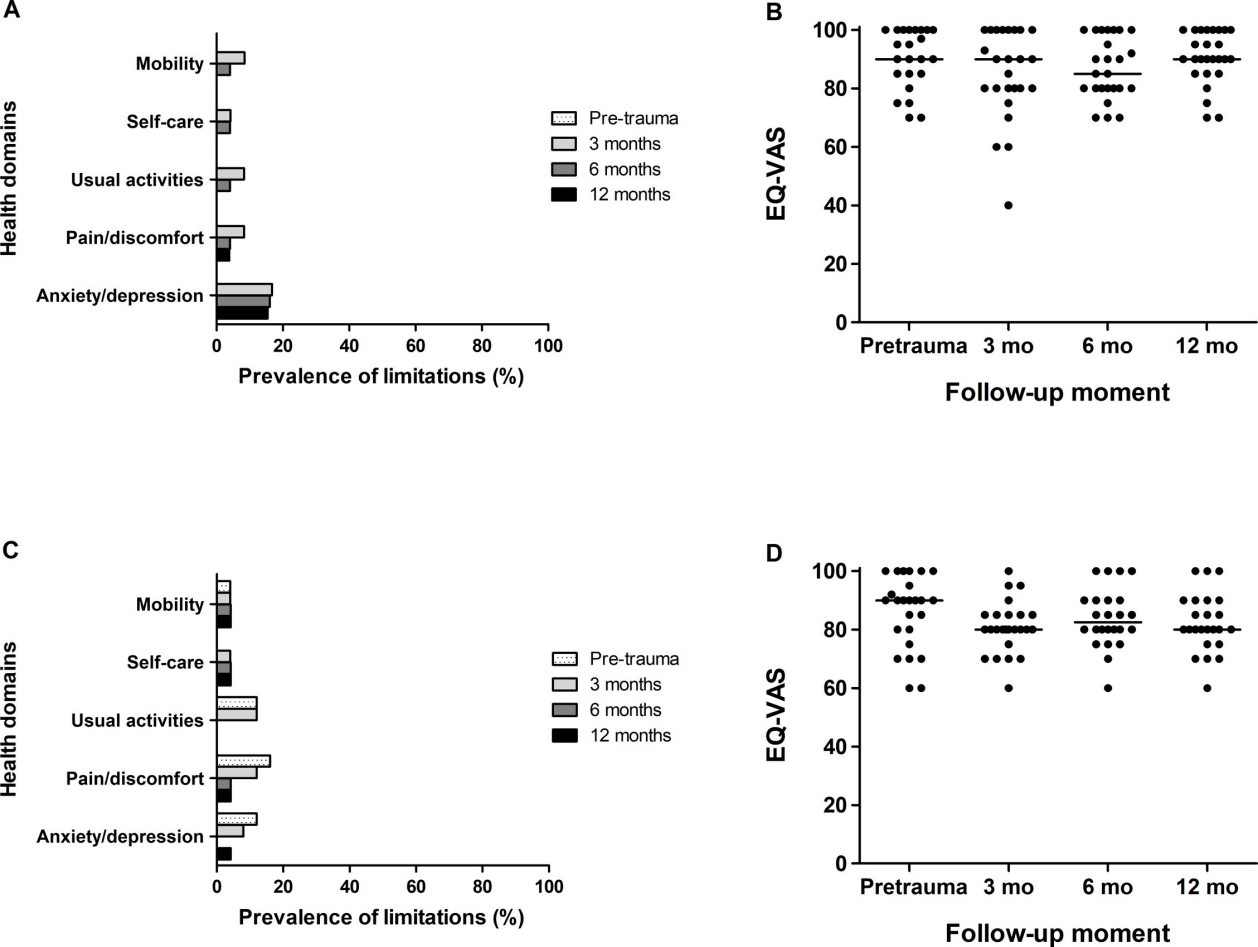
Changes over time in the EQ-5D, for patients with fireworks-related injuries. The percentage of patients who reported moderate or severe limitations on the EQ-5D (A and C) and the self-rated health status on the EQ-5D-VAS (C and D), for children (A and B) and adults (C and D). Each dot represents the score for an individual patient and the horizontal line the median score.EQ-5D, EuroQol-5D-3L; EQ-5D-VAS, EuroQol-5D-3L visual analog score; mo, months.

During follow-up patients reported their functional outcome with several questionnaires. Regarding the EQ-5D, four children reported to have complaints (little to very much) in the health domain ‘anxiety/depression’ at 6 and 12 months (Fig 3). One child reported limitations in the domain ‘pain/discomfort’ at 12 months. The median EQ-5D-VAS score was centered around 90 points at 12 months follow-up and did not differ significantly to the pre-trauma score. At 12 months, one adult reported limitations in all domains, except for ‘usual activities’. The median EQ-5D-VAS score for adults was 80 at 12 months follow-up.

Snellen visual acuity was measured in 15 out of 16 patients with eye trauma. At 12 months, six of the 13 patients with unilateral eye injury had recovered to normal vision (vision >0.8) and six patients had permanent loss of vision (vision <0.8), of which two had one legally blind eye (vision 0–0.1). Three more patients had bilateral eye injury, of which two patients fully recovered in both eyes and the other had one eye that fully recovered and one that was legally blind. One adult reported that glasses were needed to clearly see and reed (HUI-3 vision). All other patients experienced no change in their ability to see and read compared with pre-trauma.

**Fig 4:**
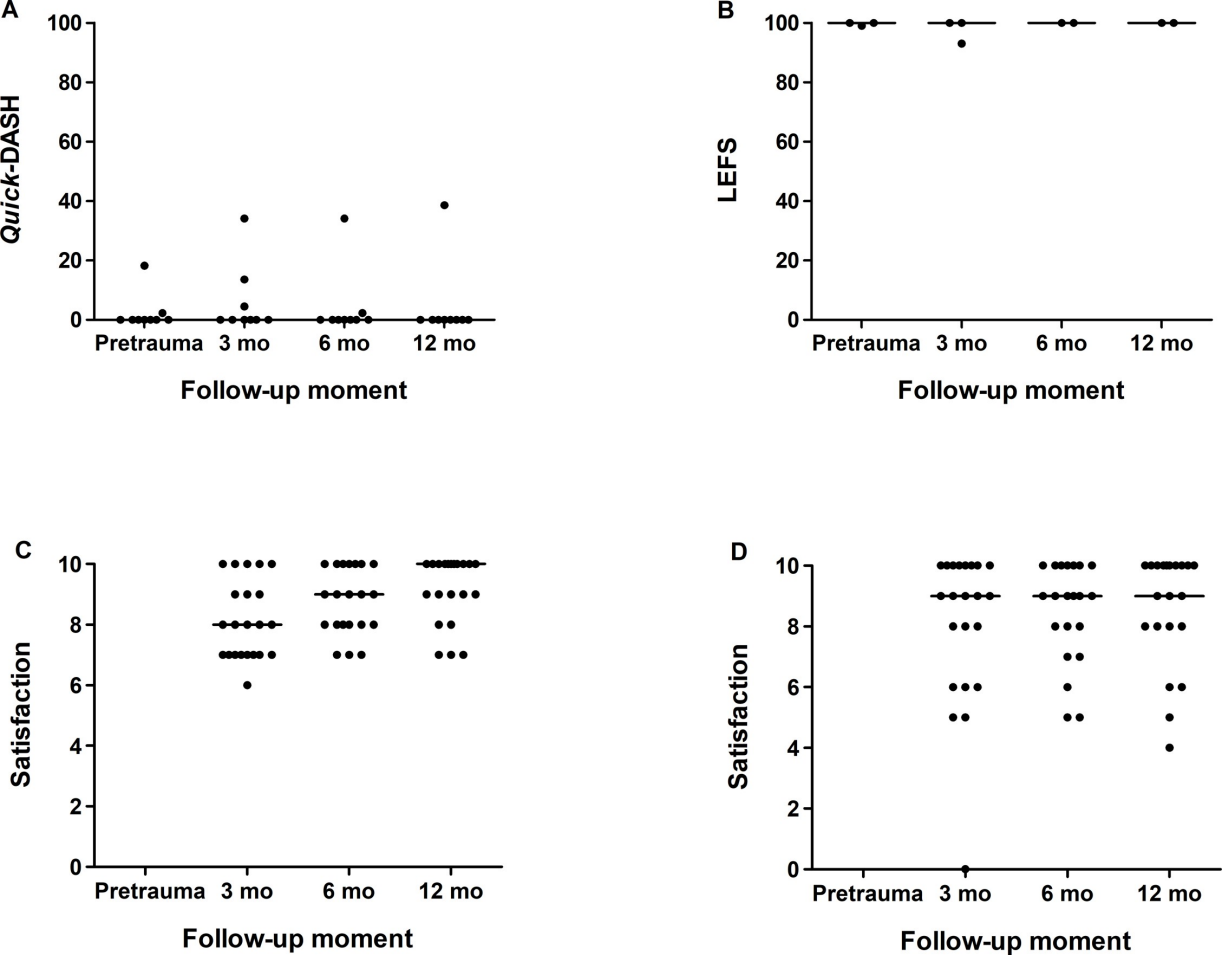
Changes over time in the Quick-DASH score (A), LEFS score (B), satisfaction with function (C) and with cosmesis (D), for all adults patients with fireworks-related injuries. Each dot represents the score for an individual patient and the horizontal line the median score. *Quick*-DASH, Quick Disabilities of the Arm, Shoulder and Hand; LEFS, Lower Extremity Functional Scale; mo, months.

At final follow-up, no patients with upper extremity injuries had problems in using their hands or fingers (HUI-3 dexterity). *Quick*-DASH scores are presented in Fig 4, along with the LEFS score for patients with lower extremity injury. Only one patient experienced limitations in upper extremity function.

Patients’ median satisfaction score regarding function and cosmesis after injury was eight or higher at all follow-up moments.

**Fig 5:**
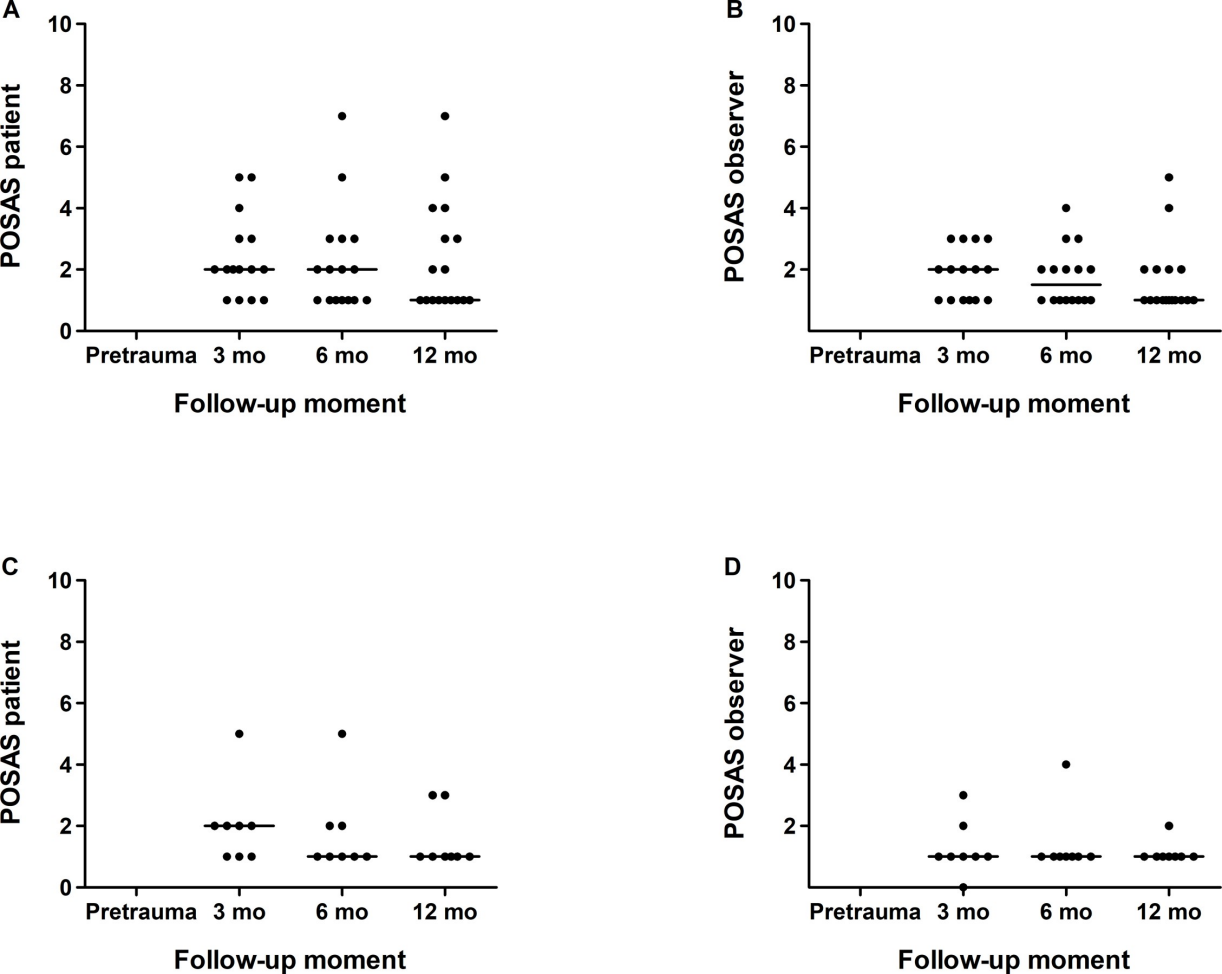
Changes over time in POSAS patient score (A and C) and POSAS observer score (B and D), for children (A and B) and adults (C and D) with fireworks-related injuries. Each dot represents the score for an individual patient and the horizontal line the median score. POSAS, patient and observer scar assessment scale; mo, months.

POSAS scores for children and adults are presented in Fig 5. Median POSAS patient score for children and adults was two or less at all follow-up moments. The POSAS observer score showed similar results, with a median score of one for both groups at 12 months.

Nineteen adults completed the questionnaire regarding their view on the use of consumer fireworks. Six patients filled that they will not lit fireworks anymore in the future. Another six patients advocate for a full ban on consumer fireworks since their injuries. Fourteen patients prefer fireworks free-zones in their neighborhood.

## Discussion

Every year, many people require hospital treatment during New Year's Eve in the Netherlands for fireworks-related injuries. These injuries sometimes have deadly consequences [2]. This study aimed to provide more information about the injuries caused by consumer fireworks, treatment needed, associated medical and societal costs, and outcome until one year. The majority of injuries appeared to occur in males and half of the patients were children or bystanders. Fireworks predominantly resulted in partial thickness burns located to upper extremity, and eye injuries. The mean total costs were €6,320 per patient, and €4,210 and €8,430 for children and adults, respectively. Only few patients experienced reduced quality of life and functional limitations after one year.

The main strength of this study is the prospective design and follow-up period of one year. Previous studies are limited to a retrospective design without follow-up. Therefore, this study was able to provide a complete overview of fireworks-related injuries and its long-term consequences, including the associated direct and indirect costs and clinical and functional outcome. Another strength of this study is the multicenter design. By including all hospitals from an entire trauma region that represents the entire country in terms of available trauma care and demographic composition, a representative sample for the entire country was obtained.

This study has some limitations. A possible flaw is that this study included only patients during one annual period (2017–2018). The number and severity of injuries from fireworks vary a lot from year to year, presumably due to the weather conditions. During the New Year’s Eve studied, it was cold and rainy. It explains why patients included in this study had less severe injuries compared with other years. This might give an underestimation of the true effects of fireworks. However, a prospective follow-up study covering multiple years was not feasible due to the limitations of the study budget. Furthermore, eight patients (13%) did not wish to participate in this study, possibly because they felt guilty or ashamed and did not want to be remembered to their injuries. Because of the relatively low number of patients this cohort might be too heterogeneous. Possibly the more severe injuries that occurred in other regions were therefore not captured in this study. However, in 2019–2020 only 385 patients were presented to a Dutch hospital with fireworks injuries [18], so including significantly more patients was not possible. We have no argument though to assume that this sample is not representative for the entire Dutch population. Another shortcoming is that only patients visiting the ER departments of hospital were registered. Undoubtedly, only the top of the iceberg was studies since much more people must have visited general practitioners with self-limiting injuries, not resulting in any permanent disability. Since we aimed to focus on the long-term effect, the short term costs are presumably underestimated. A final limitation is that the relatively low sample size did not allow subgroup analysis such as between legal and non-legal fireworks.

This study shows that the majority of injuries are located to the upper extremity (48%) and eyes (30%), which is in line with other studies [5, 19–21]. Hands and eyes are the most vulnerable body parts because they are used directly when igniting fireworks and are body parts left uncovered by any clothing.

More than 80% of the burns were of superficial depth and most eye injuries were caused by a blunt acting force. These kind of injuries might be prevented easily, for instance by wearing protective clothing, such as fire resistant gloves, or by wearing safety glasses. Also wearing appropriate clothing can help. Loose and highly flammable clothing with hoodies can easily catch fire, or fireworks can get stuck in it. Safety glasses are provided free of charge when purchasing fireworks in the Netherlands. The effectiveness of wearing safety glasses has not been demonstrated explicitly in the literature yet, but in this study none of the patients with eye injury wore any.

The vast majority of patients in this study were males and 50% concerned children below 16 years of age. This is similar to results demonstrated by other studies [4, 5, 7, 22–25]. This overrepresentation of males suggests that males tend to show more risk-taking behavior than females when using fireworks. Children in general might not acknowledge the potential dangers of fireworks and may not be capable of using it in a safe and responsible manner. Allowing children to ignite fireworks without supervision of an elderly therefore is questionable and perhaps should be forbidden.

A significant part of the patients were bystanders, which is in line with other research performed in patient with fireworks-related eye injuries [6, 19, 26]. Fireworks is often lit in crowded areas with little distance to the people spectating. This puts them at risk for being hit by diverging fireworks or flying around debris. Furthermore, fireworks might also elicits dangerous behavior, such as throwing or aiming fireworks to others, resulting in more bystanders injured.

With 2.5 million inhabitants in the trauma region where this study was performed, the total costs of fireworks-related injuries in this region were approximately €400,000 (based on 63 patients). With 17.1 million inhabitants, the total costs nationwide are estimated at €2.7 million (for 430 patients). This is comparable to €3.2 million reported by the Dutch Consumer Safety Institute [2]. These costs are withdrawn from public budgets and should be put into perspective to the €68–70 million that consumers spend annually on buying fireworks.

Due to the high number of children and bystanders injured, the public debate arises to what extent it is acceptable that people are exposed to the devastating and potential lethal consequences of fireworks when celebrating a national tradition. The Dutch government plays an important role in this debate and preventive measures should focus on young children as well. Restrictive legislation has shown to be effective in reducing the number of ocular fireworks-related injuries but for now [19], the Dutch government is against a (partial) ban of consumer fireworks.

This is the first prospective study that provides information about the functional and clinical outcome one year after an injury caused by fireworks. No data is available about the long-term outcome. In general patients in this study scored well on the patient-reported outcome scores during all follow-up moments. Self-reported quality of life (EQ-5D-VAS) was not significantly different from pre-trauma scores nor were the injury specific questionnaires. In some cases the injuries led to permanent damage, such as blind eyes or scars, with Whole Body Impairment up to 25% for a blind eye according to the American Medical Association Guides [27]. On average this did not have major or long-lasting effects on patient’s self-reported quality of life or self-reported functional abilities. Perhaps because the severity of fireworks injuries varies widely per year, or because of the heterogeneity of this cohort, this study found less severe injuries that had only limited effects on patients. Another possibility is that young patients have a great ability to cope with their injuries, or that the instruments used were not specific enough the measure functional limitations. Despite these injuries on average have no major impact on a patient’s life, they may determine their possibilities and future plans, in particular in young patients.

Unfortunately, on average 1 persons dies each year from using fireworks [2]. Also this year a person died from using fireworks [28], but it was not measured in our study because it did not occur in the study region.

## Conclusion

This study found that young males are the most vulnerable group for fireworks injuries, and that the injuries mainly consist of eye injuries and burns, mostly located to the arm and hand,. It also showed that fireworks can cause severe injuries, for which 15 (28%) patients needed hospital admission and 11 (20%) patients needed surgical treatment. Although these injuries resulted in permanent physical damage, such as scars and blind eyes, on the long term only few patients are bothered by reduced quality of life and functional limitations.

## Supporting information

S1 Table Sources and unit costs of health care resources. (PDF)

S2 Table Medication prices. (PDF)

S1 File Study protocol ROCKET study. (PDF)

S2 File STROBE checklist. (PDF)
