# Supplementary material for: Epidemiology, treatment, costs, and long-term outcomes of patients with fireworks-related injuries (ROCKET); a multicenter prospective observational case series
Source: PLoS One. 2020 Mar 19;15(3):e0230382. doi: 10.1371/journal.pone.0230382 (PMC7082032; doi:10.1371/journal.pone.0230382)
Supplement: S1 Table — (PDF) [file pone.0230382.s001.pdf]

Supplemental Table S1. Sources and unit costs of health care resources

Cost categories	Unit	Source of consumption data	Source of value	Unit price (in €)
**Medical costs**				
**Intramural**				
Transport & emergency department				
Own transportation	Ride	Study registry (CRF)	Cost manual[a]	4.51
Ambulance	Ride	Study registry (CRF)	NZa[b]	702.00
HEMS-assistance	Yes	Study registry (CRF)	Hospital data calculation[c]	4,638.00
Emergency department	Visit	Study registry (CRF)	Cost manual[a]	269.50
Surgery				
Operation room[d]	Hours	Hospital registry	Hospital[e]	729.97
Surgeon	Hours	Hospital registry	Cost manual[a]	117.58/120.70[f]
Hardware				
Titanium Elastic Nail	Per piece	Hospital registry	Industry data[g]	76.31
T-plate	Per piece	Hospital registry	Industry data[g]	52.70
Hospital stay				

General hospital	Days	Hospital registry	Cost manual[a]	460.96
Academic hospital	Days	Hospital registry	Cost manual[a]	668.03
Burn center	Days	Hospital registry	Hop et al. 2016 [1]	1,021.20
Eye hospital	Days	Hospital registry	Hospital data[h]	18,97.00/3,313.00/8,805.00[i]
Intensive Care Unit	Days	Hospital registry	Hospital data[j]	3,195.02[k]
Outpatient clinic visits				
General hospital	Visits	Hospital registry	Cost manual[a]	83.24
Academic hospital	Visits	Hospital registry	Cost manual[a]	169.61
Burn center	Visits	Hospital registry	Hospital data[j]	147.09
Eye hospital	Visits	Hospital registry	Hospital data[h]	88.00
Hand therapist	Visits	Hospital registry	Hospital data[j]	40.31
Edema therapist	Visits	Hospital registry	Hospital data[j]	40.31
**Extramural**				
General practitioner	Visits	Patient questionnaire[l]	Cost manual[a]	34.34
Physical therapist	Visits	Patient questionnaire[l]	Cost manual[a]	34.34
Occupational therapist	Visits	Patient questionnaire[l]	Cost manual[a]	34.34

Hand therapist	Visits	Patient questionnaire[l]	Cost manual[a]	34.34
Psychologist	Visits	Patient questionnaire[l]	Cost manual[a]	34.34
Visio eye rehabilitation	Visits	Patient questionnaire[l]	Cost manual[a]	66.59
Social worker	Visits	Patient questionnaire[l]	Cost manual[a]	67.63
Company doctor	Visits	Patient questionnaire[l]	Cost manual[a]	54.11
Medication	Dose per day	Hospital registry/ patient questionnaire[l]	ZiN[m]	Variable
**Work absence**				
Wage	Hour	N.A.	Cost manual[a]	39.44/32.99[n]

Reference unit prices are adjusted to 2018 by using the national consumer price index.

CRF, Case Report Form.

N.A., Not applicable.

[a] Costing manual: Methodology of costing research and reference prices for economic evaluations in healthcare, version 2016 [2].

[b] NZa, Nederlandse Zorgautoriteit [in English: Dutch Healthcare Authority): Standard costs prices, online available on: https://puc.overheid.nl/nza/doc/PUC_6237_22/.

[c] HEMS, Helicopter Emergency Medical Service: based on costs hospital 2003-2006

[d] Including operating room personnel and overhead costs.

[e] The Eye hospital provided a complete calculation of the surgery costs. These costs were used. For all other hospitals the surgery costs were calculated by multiplying the sum of the overhead costs and the hourly wage of the surgeon with the gross operation time.

[f] Hourly wage for respectively an academic and non-academic surgeon.

[g] Hardware costs were provided by the applicable firm.

[h] The Eye Hospital Rotterdam: Passanten prijslijst 2018, online available on: https://www.oogziekenhuis.nl/system/files/inline/Passanten%202018%20website%20v1.4.pdf.

[i] Admission for respectively 1-2 days, 3-5 days, or >5 days.

[j] Maasstad Hospital: Passanten prijslijst 2018, online available on: https://www.maasstadziekenhuis.nl/media/5958/passanten-01-01-2018-31-12-2018_v2.pdf.

[k] ICU admission for Burn Center Maasstad Hospital only.

[l] A customized version of the iMTA Questionnaires for the measurement of costs in economic evaluations (based on the Medical Consumption Questionnaire and Productivity Cost Questionnaire) [3, 4].

[m] ZiN = Zorginstituut Nederland [in English: National Healthcare Institute] – Standard prices were used, online available on: www.medicijnkosten.nl.

[n] National average hourly wage for respectively males and females.

References

1. Hop MJ, Wijnen BF, Nieuwenhuis MK, Dokter J, Middelkoop E, Polinder S, et al. Economic burden of burn injuries in the Netherlands: A 3 months follow-up study. Injury. 2016;47(1):203-10.
2. Hakkaart-van Roijen L, Van der Linden N, Bouwmans C, Kanters T, Tan SS. Costing manual: Methodology of costing research and reference prices for economic evaluations in healthcare [in Dutch: Kostenhandleiding: Methodologie van kostenonderzoek en referentieprijzen voor economische evaluaties in de gezondheidszorg]. 2016.
3. Questionnaires for the measurement of costs in economic evaluations: institute for Medical Technology Assessment; [Available from: https://www.imta.nl/questionnaires.
4. Bouwmans C, Krol M, Brouwer W, Severens JL, Koopmanschap MA, Hakkaart L. IMTA Productivity Cost Questionnaire (IPCQ). Value Health. 2014;17(7):A550.
