# Supplementary material for: Epidemiology, treatment, costs, and long-term outcomes of patients with fireworks-related injuries (ROCKET); a multicenter prospective observational case series
Source: PLoS One. 2020 Mar 19;15(3):e0230382. doi: 10.1371/journal.pone.0230382 (PMC7082032; doi:10.1371/journal.pone.0230382)
Supplement: S2 Table — (PDF) [file pone.0230382.s002.pdf]

Supplemental Table S2. Medication prices

Medication name	ATC code	Price (in €)
Alphagan, eye drops (2 mg/mL, 5 mL)	S01EA05	6.55
Aspirine C, tablet (650 mg)	N02BA51	3.06
Atropine, eye drops (5 mg/mL; 10 mL)	S01FA01	22.41
Augmentin, tablet (625 mg)	J01CR02	2.01
Bapscar care, cream (20 g)	N.A.	40.99
Chlooramfenicol, eye drops (0,4%; 10 mL)	S01AA01	3.53
Chlooramfenicol, ointment (10 mg/gr; 5 g)	S01AA01	2.89
Cotrimoxazol, tablet (960 mg)	J01EE01	0.43
Dexamethason, eye drops (1 mg/mL; 5mL)	S01BA01	2.72
Diclofenac, tablet (25 mg/50 mg)	M01AB05	0.39/0.07
Cosopt, eye drops (20/5 mg/mL; 0,2 mL)	S01ED51	22.60
Dura tears, eye drops (15 mL)	S01XA20	4.22
Fenistil, drops (1 mg/mL; 20 mL)	R06AB03	2.68
Flucloxacilline, tablet (500 mg)	J01CF05	0.62
Hylan, eye drops (0,15/0,15 mg/mL; 4,2 mL)	S01XA20	1.65
Hypromellose, eye drops (3,2 mg/mL; 0,6 mL)	S01XA20	0.18
Iopidine, eye drops (10 mg/mL; 25 mL)	S01EA03	5.02
Monoprost, drops (50 µg/mL; 2,5 mL)	S01EE01	4.49
Paracetamol, tablet (500 mg/1000 mg)	N02BE01	0.17/0.21
Prednisolon Minims, eye drops (10 mg/mL; 5 mL)	S01BA04	2.72
STB wounddressing, cream (15 g)	N,A,	23.02
Terra-Cotril, ointment (3,5 g)	S01CA03	2.87

Tobradex ointment (3,5 g)	S01CA01	4.65
Tobradex, eye drops (5 mL)	S01CA01	5.34
Trafloxal, eye drops (3 mg/mL; 2 mL)	S01AE01	0.28
Trafloxal, ointment (3 mg/g; 3 g)	S01AE01	4.15
Ultracortenol, ointment (5 mg/g; 5 g)	S01BA04	8.73
Vidisic Carbogel, drops (2 mg/g, 10 g)	S01XA20	3.21

ATC code, Anatomical Therapeutic Chemical Classification System.

N.A., Not applicable.

Medication costs were calculated using standard medication prices as described by the ZiN (Zorginstituut Nederland [in English: National Healthcare Institute]), online available on www,medicijnkosten,nl.

The total medication price was calculated by multiplying the price per tablet by the number of tablets used per day and the number of days that the medication was used.
