# Supplementary material for: Epidemiology, treatment, costs, and long-term outcomes of patients with fireworks-related injuries (ROCKET); a multicenter prospective observational case series
Source: PLoS One. 2020 Mar 19;15(3):e0230382. doi: 10.1371/journal.pone.0230382 (PMC7082032; doi:10.1371/journal.pone.0230382)
Supplement: S1 File — (PDF) [file pone.0230382.s003.pdf]

Epidemiology, treatment (costs) and long-term outcomes of patients with fireworks-related injuries (ROCKET); a multicenter prospective observational case series

STUDY PROTOCOL

(September 2017)

PROTOCOL TITLE 'Epidemiology, treatment (costs) and long-term outcomes of patients with firework-related injuries; a multicenter prospective observational study'

**Protocol ID**	**ROCKET**
**Short title**	**Outcomes of fireworks-related injuries**
**EudraCT number**	**N.A.**
**Version**	**1.0**
**Date**	**August, 30 2017**
**Coordinating investigator**	**Drs. D.T. Van Yperen**<br>**Maasstad Ziekenhuis**<br>**Burn Center, dept. of Surgery**<br>**Mail: yperend@maasstadziekenhuis.nl**<br>**Erasmus MC, University Medical Center Rotterdam**<br>**Trauma Research Unit, dept. Of Surgery**<br>**Mail: d.vanyperen@erasmusmc.nl**
**Project leaders**	**Dr. C.H. Van der Vlies**<br>**Maasstad Ziekenhuis**<br>**Burn Center, dept. of Surgery**<br>**Mail: vliesc@maasstadziekenhuis.nl**<br>**Dr. E.M.M. Van Lieshout**<br>**Erasmus MC, University Medical Center Rotterdam**<br>**Trauma Research Unit, dept. of Surgery**<br>**Mail: e.vanlieshout@erasmusmc.nl**
**Principal investigator(s) (in Dutch: hoofdonderzoeker/ uitvoerder)**	**Prof.dr. M.H.J. Verhofstad**<br>**Erasmus MC, University Medical Center Rotterdam**<br>**Trauma Research Unit, dept. of Surgery**<br>**P.O. Box 2040, 3000 CA Rotterdam, The Netherlands**<br>**Mail: m.verhofstad@erasmusmc.nl**
**Multicenter research: per site**	**Trauma Region Southwest Netherlands:**<br>- **Admiraal de Ruyter Ziekenhuis, Goes**<br>- **Albert Schweitzer Ziekenhuis, Dordrecht**<br>- **Beatrix Ziekenhuis, Gorinchem**<br>- **Erasmus MC, Rotterdam**<br>- **Ikazia Ziekenhuis, Rotterdam**<br>- **Maasstad Ziekenhuis, Rotterdam**<br>- **Oogziekenhuis, Rotterdam**<br>- **Franciscus Gasthuis & Vlietland, Rotterdam**<br>- **Spijkenisse Medisch Centrum, Spijkenisse**<br>- **Van Weel Bethesda Ziekenhuis, Dirksland**<br>- **IJsselland Ziekenhuis, Capelle a/d IJssel**<br>- **Zorgsaam Zeeuws Vlaanderen, Terneuzen**
**Sponsor (in Dutch: verrichter/opdrachtgever)**	**Erasmus MC, University Medical Center Rotterdam**<br>**Trauma Research Unit, dept. of Surgery**<br>**P.O. Box 2040, 3000 CA Rotterdam, The Netherlands**
**Subsidising party**	**Not applicable**
**Independent expert (s)**	**Not applicable**
**Laboratory sites**	**Not applicable**
**Pharmacy**	**Not applicable**

PROTOCOL SIGNATURE SHEET

Name	Signature	Date
Sponsor or legal representative:<br>Erasmus MC<br><br>Head of Department:<br>Prof. dr. J.M. Hendriks, surgeon		
Principal Investigator:<br><br>Prof.dr. M.H.J. Verhofstad<br>Trauma surgeon		
Project Leader:<br><br>Dr. C.H. Van der Vlies<br>Trauma surgeon		
Project Leader:<br><br>Dr. E.M.M. Van Lieshout<br>Research coordinator Trauma Surgery		

PROTOCOL SIGNATURE SHEET

Name	Signature	Date
**Sponsor or legal representative:**<br>Erasmus MC<br><br>Head of Department:<br>Prof. dr. J.M. Hendriks, surgeon		12-09-2017
**Principal Investigator:**<br><br>Prof.dr. M.H.J. Verhofstad<br>Trauma surgeon		15-9-17
**Project Leader:**<br><br>Dr. C.H. Van der Vlies<br>Trauma surgeon		12-09-2017
**Project Leader:**<br><br>Dr. E.M.M. Van Lieshout<br>Research coordinator Trauma Surgery		12-09-2017

TABLE OF CONTENTS

1. INTRODUCTION AND RATIONALE 8
2. OBJECTIVES 10
3. STUDY DESIGN 11
4. STUDY POPULATION 12
 4.1 Population (base) 12
 4.2 Inclusion criteria 12
 4.3 Exclusion criteria 12
 4.4 Sample size calculation 12
5. TREATMENT OF SUBJECTS 13
 5.1 Investigational product/treatment 13
 5.2 Use of co-intervention (if applicable) 13
 5.3 Escape medication (if applicable) 13
6. INVESTIGATIONAL PRODUCT 14
 6.1 Name and description of investigational product(s) 14
 6.2 Summary of findings from non-clinical studies 14
 6.3 Summary of findings from clinical studies 14
 6.4 Summary of known and potential risks and benefits 14
 6.5 Description and justification of route of administration and dosage 14
 6.6 Dosages, dosage modifications and method of administration 14
 6.7 Preparation and labelling of Investigational Medicinal Product 14
 6.8 Drug accountability 14
7. NON-INVESTIGATIONAL PRODUCT 15
 7.1 Name and description of non-investigational product(s) 15
 7.2 Summary of findings from non-clinical studies 15
 7.3 Summary of findings from clinical studies 15
 7.4 Summary of known and potential risks and benefits 15
 7.5 Description and justification of route of administration and dosage 15
 7.6 Dosages, dosage modifications and method of administration 15
 7.7 Preparation and labelling of Non Investigational Medicinal Product 15
 7.8 Drug accountability 15
8. METHODS 16
 8.1 Study parameters/endpoints 16
  8.1.1 Main study parameter/endpoint 16
  8.1.2 Secondary study parameters/endpoints (if applicable) 17
  8.1.3 Other study parameters (if applicable) 20
 8.2 Randomization, blinding and treatment allocation 21
 8.3 Study procedures 21
 8.4 Withdrawal of individual subjects 23
  8.4.1 Specific criteria for withdrawal (if applicable) 23
 8.5 Replacement of individual subjects after withdrawal 23
 8.6 Follow-up of subjects withdrawn from treatment 23

8.7 Premature termination of the study 23
9. SAFETY REPORTING 24
9.1 Temporary halt for reasons of subject safety 24
9.2 AEs, SAEs and SUSARs 24
9.2.1 Adverse events (AEs) 24
9.2.2 Serious adverse events (SAEs) 24
9.2.3 Suspected unexpected serious adverse reactions (SUSARs) 24
9.3 Annual safety report 24
9.4 Follow-up of adverse events 24
9.5 Data Safety Monitoring Board (DSMB) / Safety Committee 24
10. STATISTICAL ANALYSIS 25
10.1 Primary study parameter(s) 25
10.2 Secondary study parameter(s) 25
10.3 Other study parameters 25
10.4 Interim analysis (if applicable) 25
11. ETHICAL CONSIDERATIONS 26
11.1 Regulation statement 26
11.2 Recruitment and consent 26
11.3 Objection by minors or incapacitated subjects (if applicable) 26
11.4 Benefits and risks assessment 27
11.5 Compensation for injury 27
11.6 Incentives (if applicable) 28
12. ADMINISTRATIVE ASPECTS, MONITORING AND PUBLICATION 29
12.1 Handling and storage of data and documents 29
12.2 Monitoring and Quality Assurance 29
12.3 Amendments 30
12.4 Annual progress report 30
12.5 Temporary halt and (prematurely) end of study report 30
12.6 Public disclosure and publication policy 30
13. STRUCTURED RISK ANALYSIS 31
13.1 Potential issues of concern 31
13.2 Synthesis 31
14. REFERENCES 32

LIST OF ABBREVIATIONS AND RELEVANT DEFINITIONS

95% CI	95% confidence interval
AE	Adverse Event
CISS	Cold Intolerance Symptoms Severity score
CPSC	Consumer Product Safety Commission
CRF	Case Report Form
DSMB	Data Safety Monitoring Board
ED	Emergency Department
EQ-5D-3L	EuroQoL-5D-3L
EudraCT	European drug regulatory affairs Clinical Trials
GP	General Practitioner
HEMS	Helicopter Emergency Medical Services
HUI-3	Health Utilities Index
ICU	Intensive Care Unit
LEFS	Lower Extremity Functional Scale
METC	Medisch Ethische Toetsing Commissie (METC); in English: Medical Research Ethics Committee (MREC)
MREC	Medical Research Ethics committee (MREC); in Dutch: Medisch Ethische Toetsing Commissie (METC)
NTR	Nederlands Trial Register (in English; Netherlands National Trial Register)
POSAS	Patient and Observer Scar Assessment Scale
Quick-DASH	Quick Disabilities of Arm, Shoulder and Hand score
SD	Standard Deviation
Sponsor	The sponsor is the party that commissions the organisation or performance of the research, for example a pharmaceutical company, academic hospital, scientific organisation or investigator. A party that provides funding for a study but does not commission it is not regarded as the sponsor, but referred to as a subsidising party.
SPSS	Statistical Package for the Social Sciences
STROBE	STrengthening the Reporting of OBservational studies in Epidemiology
SUSAR	Suspected Unexpected Serious Adverse Reaction
TBSA	Total Body Surface Area
VAS	Visual Analogue Score
Wbp	Personal Data Protection Act (in Dutch: Wet Bescherming Persoonsgevens)
WMO	Medical Research Involving Human Subjects Act (in Dutch: Wet Medisch-wetenschappelijk Onderzoek met Mensen

SUMMARY

Rationale: Fireworks-related injuries frequently occur in both adults and children. It often entails serious and avoidable injuries to innocent bystanders. Most injuries are non-fatal but a substantial amount results in life-long disabilities affecting quality of life and the use of public health services. Currently, reliable data about the affected patients, their injury characteristics, the direct and indirect costs resulting from their injury and the loss of quality of life outcomes in the long-term is lacking.
Objective: The main aim is to provide detailed information about the injury characteristics of patients reporting to a hospital with fireworks-related injuries. Secondary aims are to determine the treatment (and associated direct medical and indirect societal costs), clinical and functional outcome.
Study design: Multicenter, prospective, observational case series
Study population: All patients (no age limit) with fireworks-related injuries occurred between December 1, 2017 and January 31, 2018 are eligible for inclusion.
Intervention (if applicable): Not applicable
Main study parameters/endpoints: The main outcome measures are the injury characteristics. The secondary outcomes are 1) the number and type of operations needed; 2) the duration of hospital admission; 3) the direct medical and indirect societal costs; 4) the absence from school; 5) the quality of life (EuroQol-5D-3L; EQ-5D-3L) and injury specific outcomes (Health Utilities Index 3; HUI-3 (domains vision and dexterity), visual acuity, *Quick* Disabilities of Arm, Shoulder and Hand (*Quick*-DASH); Cold Intolerance Severity Scale (CISS); Lower Extremity Functional Scale (LEFS); Patient and Observer Scar Assessment Scale (POSAS); 6) patient satisfaction with their functional recovery and cosmesis; and 7) how the trauma affected patient's view on the use of consumer fireworks.
Nature and extent of the burden and risks associated with participation, benefit and group relatedness: The clinical follow-up program including outpatient department visits, physical examination, and diagnostics is not influenced by this study. At three, six and 12 months after presentation, adult patients and parents of pediatric patients will be asked to complete the quality of life questionnaires and the injury specific questionnaires. The investigation and completion of questionnaires will take approximately 20 minutes per visit. No other inconvenience associated with participation is expected. The risks associated with participation can be considered negligible and the burden can be considered minimal. The study could not be conducted without the participation of pediatric patients.

1. INTRODUCTION AND RATIONALE

Fireworks are used for celebrating all kinds of rituals and events worldwide. Despite their nice visible and audible effects, it has the potential to cause serious injuries.

Fireworks-related injuries occur frequent in both adults and children. It can be considered a public health problem as they often entail serious and avoidable injuries and often affect innocent bystanders (1-3).

In the USA, the Consumer Product Safety Commission (CPSC) presented a report stating that in 2015 approximately 12,000 fireworks-related injuries were treated in Emergency Departments (ED), reflecting an annual incidence rate of 3.7 per 100,000 individuals. Most patients were males (61%) and 42% were younger than 20 years of age (4). This is comparable with the results of several other studies (1-3, 5-9).

The percentages presented by the CPSC are comparable with the Netherlands with an incidence rate of 2.8 per 100.000 individuals per year treated at an ED with fireworks-related injuries(8). In 36% of the cases, victims were younger than 20 years of age and 76% were males. In the past ten years the number of fireworks-related injuries fluctuates between 400 and 1,000 cases annually. A clear up or downward trend is not observed in the past decade (8).

In two-thirds of all patients, the injuries are located at the hands, face and eyes, mostly consisting out of burns, superficial injuries, and open wounds (2, 5-8, 10, 11).

Most firework-related injuries are non-fatal but they do have the potential to result in long-term disability (3, 5, 8, 10). This has a great effect on the individuals' quality of life and the use of public health services, with high direct medical and indirect societals costs. A Dutch report published in 2016, calculated that the direct medical costs of an individual visiting the ED/admitted to the hospital with a fireworks-related injury are on average €1.600 per individual, based on the years 2011 – 2016, with annual costs of around €1.1 million. When the indirect costs due to absence from work are included as well, this estimate rose to €8.100 per individual and to €2.9 million annually (8).

The fireworks-related injuries in the Netherlands are concentrated around the night of New Years' Eve. Every year the fireworks-related injuries are recorded by the Dutch Society of Opthalmology, Dutch Association of Plastic Surgeons and the Dutch Trauma Society. Since 2004, also a nationwide estimation on the number of fireworks-related injuries is made by the Consumer Safety Institute (2, 8). However, this number is an estimation based on a sample of Dutch hospitals. Detailed information about patient and injury characteristics, medical and societal costs and long-term functional outcomes are lacking. This kind of information aids the public debate and contributes to the political decision on whether or not to abolish the use of consumer fireworks.

Fireworks-related injuries are relatively easy to prevent and therefore a relevant public health problem. However, detailed knowledge is lacking on the treatment, functional impairment, and medical and societal costs on the long-term. This study aims to provide detailed information regarding patient, injury, and treatment characteristics, medical and societal costs, and patient-reported outcomes, with a follow-up of one year.

2. OBJECTIVES

Primary Objective:
To determine the **injury characteristics** of fireworks-related injuries in patients reporting to a hospital for treatment.

Secondary Objective(s):
1) To determine the **number and type of operations** needed due to of fireworks-related injuries until one year after trauma
2) To determine the **duration of hospital admission** in these patients
3) To determine the **direct medical costs** and the **indirect societal costs (incl. work absence)** in these patients
4) To investigate the **absence from school** in these patients
5) To investigate the **patient-reported outcomes** in these patients
    - EuroQol-5D (EQ-5D-3L)
    - Health Utility Index Mark 3 (domains for vision and dexterity) (HUI-3)
    - *Quick* Disabilities of the Arm, Shoulder and Hand (*Quick*-DASH)
    - Cold Intolerance Symptoms Severity (CISS)
    - Lower Extremity Functional Scale (LEFS)
    - Patient and Observer Scar Assessment Scale (POSAS)
    - Patient satisfaction with the functional recovery and cosmesis
6) To investigate how the injury changed **patient's view on the use of fireworks**

3. STUDY DESIGN

This study will be a multicenter, prospective, observational case series.

4. STUDY POPULATION

4.1 Population (base)

All patients (no age limit) with fireworks-related injuries presenting to a hospital in the period between December 1, 2017 and January 31, 2018 in the Trauma Region Southwest Netherlands, will be eligible for inclusion. Patients will be recruited from every hospital in the Trauma Region Southwest Netherlands. In the Netherlands it is only permitted to lit fireworks as a consumer during the celebration of New Year's Eve and logically most fireworks-related injuries occur during that night or the period close before and after. Therefore, this specific time range was chosen.

4.2 Inclusion criteria

In order to be eligible to participate in this study, a subject must meet all of the following criteria:

1) Patients with any injury caused by fireworks treated* in a hospital in the Trauma Region Southwest Netherlands between December 1, 2017 and January 31, 2018
2) Provision of informed consent

* Treatment is defined as any intervention for which ≥1 clinical follow-up visit is scheduled

4.3 Exclusion criteria

A potential subject who meets any of the following criteria will be excluded from participation in this study:

1) Patients who died within the first 24 hours due to other injuries than fireworks
2) Patients with incomplete or unknown contact information;
3) Insufficient understanding of Dutch or English to understand the study documents

4.4 Sample size calculation

A report from VeiligheidNL mentions 574 patients during the annual change of year in 2014-2015 in The Netherlands. With 2 full months for inclusion we expect to increase this by at least 10% (n=638). The Southwest Netherlands area contains 10 of the 90 Dutch hospitals. This could project to a possible sample of 70-80 patients. A formal sample size calculation for this observational study is not constructive. By including all patients treated in the Trauma Region Southwest Netherlands during the specific time interval, the largest possible sample will be enrolled. The larger the number of patients, the more reliable the analysis will be. Enrolling all patients possible will result in the highest reliability of data (*i.e.*, accurate point estimate with lowest possible variance).

5. TREATMENT OF SUBJECTS

Not applicable, this study is not an intervention study.

5.1 Investigational product/treatment

Not applicable, this study is not an intervention study.

5.2 Use of co-intervention (if applicable)

Not applicable, this study is not an intervention study.

5.3 Escape medication (if applicable)

Not applicable, this study is not an intervention study.

6. INVESTIGATIONAL PRODUCT

Not applicable, as this study does not involve an investigational product.

6.1 Name and description of investigational product(s)

Not applicable, as this study does not involve an investigational product.

6.2 Summary of findings from non-clinical studies

Not applicable, as this study does not involve an investigational product.

6.3 Summary of findings from clinical studies

Not applicable, as this study does not involve an investigational product.

6.4 Summary of known and potential risks and benefits

Not applicable, as this study does not involve an investigational product.

6.5 Description and justification of route of administration and dosage

Not applicable, as this study does not involve an investigational product.

6.6 Dosages, dosage modifications and method of administration

Not applicable, as this study does not involve an investigational product.

6.7 Preparation and labelling of Investigational Medicinal Product

Not applicable, as this study does not involve an investigational product.

6.8 Drug accountability

Not applicable, as this study does not involve an investigational product.

7. NON-INVESTIGATIONAL PRODUCT

Not applicable, as this study does not involve a non-investigational product.

7.1 Name and description of non-investigational product(s)

Not applicable, as this study does not involve a non-investigational product.

7.2 Summary of findings from non-clinical studies

Not applicable, as this study does not involve a non-investigational product.

7.3 Summary of findings from clinical studies

Not applicable, as this study does not involve a non-investigational product.

7.4 Summary of known and potential risks and benefits

Not applicable, as this study does not involve a non-investigational product.

7.5 Description and justification of route of administration and dosage

Not applicable, as this study does not involve a non-investigational product.

7.6 Dosages, dosage modifications and method of administration

Not applicable, as this study does not involve a non-investigational product.

7.7 Preparation and labelling of Non Investigational Medicinal Product

Not applicable, as this study does not involve a non-investigational product.

7.8 Drug accountability

Not applicable, as this study does not involve a non-investigational product.

8. METHODS

8.1 Study parameters/endpoints

8.1.1 Main study parameter/endpoint

The primary outcome measures are the **injury characteristics**. The following variables will be collected:

- Number of injuries
- Body site injured
  1) Head/neck (scalp, forehead, orbital, nose, cheek, mouth/lips, chin, ear, or neck)
  2) Eye
  3) Upper extremity (shoulder, upper arm, elbow, lower arm, wrist, hand palm, hand dorsum, thumb/first webspace, digit 2, 3, 4, 5, or other (text))
  4) Lower extremity (hip, upper leg, knee, lower leg, ankle, foot, other (text))
  5) Trunk (chest, abdomen, back, pelvis, or other (text))
  6) Other (text)
- Injured side (left, right, or not applicable)
- Type of injury per site (including text description)
  1) Burn (total body surface area (TBSA) and degree)
  2) Globe rupture (open or closed)
  3) Intra-ocular foreign body
  4) Penetration/perforation
  5) Corneal/adnex burn (chemical or thermic)
  6) Lens injury
  7) Retina injury
  8) Choroidal injury
  9) Corneal rupture
  10) Soft tissue damage (superficial or deep)
  11) Fracture/dislocation (fracture location)
  12) Amputation (level of amputation))
  13) Vascular damage (affected vessel)
  14) Neurological damage (affected nerve)
  15) Tendon damage (affected tendon)
  16) Other (text)

8.1.2 Secondary study parameters/endpoints (if applicable)

The following outcomes will serve as secondary outcome measures:

- Number and type of operations
- Duration of hospital admission
- Direct medical and indirect societal costs
- Absence from school
- Quality of life (EuroQol-5D-3L; EQ-5D-3L)
- Health Utilities Index 3; HUI-3 (domains vision and dexterity)
- Visual acuity
- Quick Disabilities of Arm, Shoulder and Hand (*Quick*-DASH)
- Cold Intolerance Severity Scale (CISS)
- Lower Extremity Functional Scale (LEFS)
- Patient and Observer Scar Assessment Scale (POSAS)
- Patient satisfaction with their functional recovery and cosmesis
- How trauma has affected patient's view on the use of consumer fireworks

The **number and type of operations** performed will be extracted from patients' medical file.

- Operation performed (yes or no)
- Total number of operations performed
- Type of first operation (soft tissue care, enucleation, evisceration, amputation, fracture treatment, revascularization, transplantation, reconstruction (skin grafting, flaps, or other (text description)), repair (vessels, nerves, tendons), other (text description), or multiple) (including text details)
- Indications for the second, third and any other subsequent operation (text description)

The total **duration of hospital admission** will be calculated from the dates of admission and discharge from the hospital. Re-admission to a hospital during follow-up will be counted separately.

In order to determine the **direct medical costs** and **indirect societal costs**, patients will be asked to complete a custom-made questionnaire that contains detailed information on both items. Health care costs will include general practice care, medical specialist care, nursing care, physical therapy, hospitalization,

medication, home care, and other costs directly associated with diagnosis, treatment and rehabilitation. The **indirect societal costs** will be calculated by the absence from paid work, production loss without absence from paid work and hindrance in the performance of paid and unpaid work.

School-going patients (and their parents) will be asked to complete a custom-made questionnaire regarding **absence from school**.

The **EuroQol-5D-3L (EQ-5D-3L)** is a validated questionnaire for measuring health-related quality of life (12, 13). The EQ-5D-3L descriptive system consists of five dimensions of health (mobility, self-care, usual activities, pain/discomfort, anxiety/depression), each with three possible answers. Scores are converted to a utility score ranging from zero to one, with lower scores indicating poorer quality of life. The EQ VAS records the respondents self-rated health status on a vertical (0-100) visual analog scale, which should be used together with the descriptive system to build a composite picture of the respondent's health status. Patients aged 16 years or older will complete the EQ-5D-3L themselves. Parents of pediatric patients 4-7 years old will complete the youth proxy version (EQ-5D-3L-Y proxy), and pediatrics patients between 8-15 years old will complete the ‘youth’ version (EQ-5D-3L-Y) by themselves or if necessary with the help of the parents.

For specific injuries we will ask patients to complete additional questionnaires, in order to gain more insights in injury specific outcomes.

<u>For patients with eye injuries:</u>
The **Health Utilities Index Mark 3 (HUI-3), domain vision** (consisting out of 2 questions, each with four possible answers), will be used to assess patients generic health status and health-related quality of life, regarding their eye injury. Patients aged ≥16 years will complete the HUI-3 themselves. Parents of a pediatric patient (<16 years) will complete the questionnaire, together with their child if possible.

Visual acuity is determined routinely and will be extracted from patient’s medical file.

<u>For patients with upper extremity injuries:</u>
The **HUI-3, domain dexterity** (one question with four possible answers) will assess the health-related quality of life regarding upper extremity injuries. Patients aged ≥16

years will complete the HUI-3 themselves. Parents of a pediatric patient (<16 years) will complete the questionnaire, together with their child if possible.

The ***Quick*-DASH** is used in order to measure physical function and symptoms in people with any or multiple musculoskeletal disorders in the upper limb. It is scored in two components: the disability/symptom section (11 items, scored 1-5) and the optional high performance sport/music or work modules (each 4 items, scored 1-5). A higher score indicates greater disability (14). Only patients aged ≥16 years will complete this questionnaire.

The **Cold Intolerance Symptoms Severity (CISS)** questionnaire is a 6-item questionnaire. The sum of all questions (range 4-100) defines the severity of cold intolerance, with a higher number representing more severe symptoms (15, 16). Only patients aged ≥16 year will complete this questionnaire.

For patients with lower extremity injury:
The **Lower Extremity Functional Scale (LEFS)** is a validated questionnaire containing 20 items about a person's ability to perform everyday tasks. It can be used to evaluate the functional impairment of a patient with a disorder of one or both lower extremities. All items are scored so that a high score represents greater disability. The maximum score achievable is 80 points (17, 18). Only patients aged ≥16 year will complete this questionnaire.

For patients with burn wounds
The **Patient and Observer Scar Assessment Scale** (POSAS v2.0; www.posas.org) (19) consists of two parts: a Patient Scale and an Observer Scale, which aim to provide a rating of several measured of scar quality (vascularity, pigmentation, relief/texture, thickness, pliability, surface area, pain, and itching/pruritus). Adult patients and parents of pediatric patients (<16 years) will complete the patient-reported part. A trained observer will complete the observer-reported part.

Satisfaction level regarding function and cosmesis of the injured area will be determined using an 11-point numeric rating scale, ranging from 0 (extremely dissatisfied) to 10 (fully satisfied). Only patients aged ≥16 year will complete this questionnaire.

Patients will also be asked to complete a custom-made questionnaire investigating their **view on the use of consumer fireworks** before and after trauma, and how the trauma influenced their opinion. Only patients aged ≥16 year will complete this questionnaire.

8.1.3 Other study parameters (if applicable)

In order to report characteristics of the study population and to determine treatment costs, patient characteristics, additional injury-related and outcomes-related variables will be collected. These will include:

Patients characteristics to be collected:

- Age (calculated from date of birth and date of treatment)
- Gender (male or female)
- Medical history (diabetes, Raynaud's disease, rheumatoid arthritis, peripheral nerve injury, vascular diseases, or other (text))
- American Society of Anesthesiologists (ASA) classification (ASA 1, 2, 3, 4, 5, or unknown)
- Pre-injury occupation (work, hobby, sports) (text description) and hours spent per week (text))
- Hand dominance (left or right; for patients with upper extremity injuries only)
- Eye dominance (left, right, unknown; for patients with eye injuries only)

Additional injury characteristics to be collected:

- Injury occurred within the permitted period to set off fireworks (determined by the date and time of injury and the legally permitted period to set off fireworks)
- Type of fireworks (sparklers, firecrackers, roman candles, rockets, shells/mortars, carbid, homemade, other (text), or unknown)
- Role (igniter, bystander, other, or unknown)
- Legal fireworks (yes, no, or unknown)
- Correct use of fireworks (yes, no, or unknown)
- Setting of injury (domestic, work related, professional firework display, other (text), or unknown)

Treatment characteristics to be collected:

- Prehospital transport (ambulance, Helicopter Emergency Medical Services (HEMS), self-transport, other (text), or unknown)

- HEMS assistance (yes, no, or unknown)
- Referral to hospital (direct or indirect (general practitioner, general practice center, other hospital, or other (text)))
- Admission (yes or no)
- Required care (ward, Intensive Care Unit (ICU), observatory, or other)
- Wound treatment (wound dressings (text), topical therapy (text), other (text), unknown, or not needed)
- Number of transfusions required (packed-cells, platelets, plasma, colloid, or other (text description))
- Discharge destination (home, other hospital, nursing home, rehabilitation facility, burn center, or other)
- Need for prosthesis (yes or no, and description)

Outcome-related variables to be collected:

- Time between initial injury and hospital presentation (calculated from dates of trauma and hospital presentation)
- Duration of hospital admission (calculated from date of admission and date of discharge)
- Duration of ICU admission (in days) (calculated from dates of admission and discharge)
- Mortality (fireworks-related, yes or no)
- Rehabilitation (physical therapy, hand therapy, ergotherapy, other (text description), or multiple)
- Complications and secondary interventions (text description)

8.2 Randomization, blinding and treatment allocation

Randomization and treatment allocation are not applicable, since this study is not an intervention study. Blinding of patients and surgeons also does not apply.

8.3 Study procedures

A: Patient selection

The local contact person in the participating hospital will provide details of each patient with fireworks-related injuries to the research team. A simple e-mail or phone call is sufficient for registration, the coordinating investigator (or a dedicated and trained research assistant) will take care of all further logistics and data collection. He/she will also call each hospital on a daily basis during the inclusion period in order to ask for any

missed registrations. As a check for completeness of inclusion, hospital registries will be searched by the coordinating investigator or research assistant.

B: Data collection

Patients will be followed for one year, with the first visit for baseline measurement planned as soon as possible, preferably within two weeks. Follow-up visits are planned at three months (window 2-4 months), six months (5-7 months), and 12 months (window 12-14 months) after presentation.. In case of early discharge from clinical treatment/follow-up, subsequent follow-up can be performed by visiting patients at home.

The study will not interfere with treatment or follow-up. Data concerning patient characteristics, injury characteristics, treatment details, and clinical outcome data will be extracted from the patient file as much as possible. These data will be collected from the patient's medical files as soon as possible after signing informed consent. For the purpose of the study, additional details concerning treatment and outcome (see paragraph 8) that are not mentioned in the patient's hospital files, will be registered on the case report forms directly. This will be done at the follow-up visits.

As soon as possible and within two weeks after inclusion patients will be asked to complete the questionnaire's, reflecting their pre-injury health status. For all participants this will be the EQ-5D-3L questionnaire. For patients with eye trauma the HUI-3 (domain vision), for patients with upper extremity trauma the HUI-3 (domain dexterity), the *Quick*-DASH, and CISS, for patients with lower extremity trauma the LEFS, and for patients with burn wounds the POSAS. At three, six and 12 months, treatment characteristics, health care consumption details and patient reported outcomes will be collected.

Table 1: Follow-up schedule and data collection

**Event forms**	**Screening**	**<2 weeks** (<2 we)	**3 months** (2-4 mo)	**6 months** (5-7 mo)	**12 months** (12-14 mo)
Screening	X				
Informed Consent		X			
Patient characteristics		X			
Injury characteristics		X	X		
Radiology		X[1,2]	X[1]	X[1]	X[1]
Digital photo		X[2,3]	X[3]	X[3]	X[3]
Treatment characteristics		X	X	X	X
Outcome details		X	X	X	X
Adverse events		X	X	X	X
Health care consumption		X[2]	X	X	X

Work or school absence	X[2]	X	X	X
EQ-5D-3L	X[2]	X	X	X
HUI-3 domain vision[4]	X[2]	X	X	X
HUI-3 domain dexterity[5]	X[2]	X	X	X
Quick-DASH[5]	X[2]	X	X	X
CISS[5]	X[2]	X	X	X
LEFS[6]	X[2]	X	X	X
POSAS[7]		X	X	X
Satisfaction with functional recovery and cosmesis		X	X	X
View on fireworks			X	
Early withdrawal	*	*	*	*

[1] Only if treating physician requests radiographs; [2] Representing the pre-injury status, [3] Optional; [4] For patients with eye injury, [5] For patients with upper extremity injury, [6] For patients with lower extremity injury, [7] For patients with burn wounds. * Only at time of withdrawal
For EQ-5D and HUI-3, pediatric versions are available for children <16 years. Parents of children <16 years complete the POSAS. Other questionnaires are completed by patients ≥16 years only.

8.4 Withdrawal of individual subjects

Participants can leave the study at any time for any reason if they wish to do so without having to give a reason. Their decision to withdraw will have no consequence for their medical treatment. The investigator can decide to withdraw a subject from the study for urgent medical reasons.

8.4.1 Specific criteria for withdrawal (if applicable)

Not applicable

8.5 Replacement of individual subjects after withdrawal

Not applicable; no replacement will take place.

8.6 Follow-up of subjects withdrawn from treatment

Should a participant who is still under routine clinical follow-up decide to withdraw consent, they will receive the normal clinical follow-up as any other patient treated for fireworks-related injuries. As treatment are not part of this study, no additional controls are needed. Also, the research team will no longer contact patients after consent has been withdrawn.

8.7 Premature termination of the study

This will not occur, as treatment is not part of the study.

9. SAFETY REPORTING

As this study is not subject to the Medical Research Involving Human Subjects Act (WMO), patients are not subjected to treatment in this study, and the study does not involve an investigational or non-investigational medicinal product, this paragraph does not apply.

9.1 Temporary halt for reasons of subject safety

As patients are not subjected to treatment in this study, this does not apply.

9.2 AEs, SAEs and SUSARs

9.2.1 Adverse events (AEs)

As patients are not subjected to treatment in this study, this does not apply.

9.2.2 Serious adverse events (SAEs)

As patients are not subjected to treatment in this study, this does not apply.

9.2.3 Suspected unexpected serious adverse reactions (SUSARs)

As patients are not subjected to treatment in this study and the study does not involve an investigational medicinal product, this does not apply.

9.3 Annual safety report

As patients are not subjected to treatment in this study and the study does not involve an investigational medicinal product, this does not apply.

9.4 Follow-up of adverse events

As patients are not subjected to treatment in this study, this does not apply.

9.5 Data Safety Monitoring Board (DSMB) / Safety Committee

As patients are not subjected to treatment in this study and the study does not involve an investigational nor a non-investigational medicinal product, participants will have no larger risks than patients who are not participating in this study. Therefore, no DSMB will be installed.

10. STATISTICAL ANALYSIS

Data will be analyzed using the Statistical Package for the Social Sciences (SPSS) version 21.0 or higher (SPSS, Chicago, Ill., USA). Normality of continuous data will be tested with the Shapiro-Wilk test, and homogeneity of variances will be tested using the Levene's test. A p-value <0.05 will be taken as threshold of statistical significance in all statistical tests, and all tests will be two-sided. Missing values will not be replaced by imputation.

10.1 Primary study parameter(s)

Injury characteristics will be analyzed descriptively. They will be reported as mean with Standard Deviation (SD; parametric, continuous data) or median with quartiles (P_{25}-P_{75}; non-parametric, continuous data), or as number with percentage (categorical data).

Data will be reported as a group as well separately for the groups of patients with eye injury, upper and lower extremity injury, hand injury, or burn wounds. Further subgroups will be determined based on the patients included. Relevant subgroups can be legal versus illegal fireworks, operator versus bystander.

10.2 Secondary study parameter(s)

Secondary outcome measures will be analyzed and reported the same as described for the primary outcome measures (paragraph 10.1). The only difference is that costs will be reported as mean with 95% confidence interval (95% CI). The 95% CI around the mean costs will be approximated by nonparametric bootstrapping.

10.3 Other study parameters

See section 10.2.

In order to check if the enrolled sample is a true representation of the total group of patients treated in a hospital for fireworks-related injuries. Gender and location of injuries of participants and non-participants will be compared using a Chi-squared test or Fisher's test. Differences in age between the two groups will be tested using a Student's T-test or Mann-Whitney U-test, as applicable.

10.4 Interim analysis (if applicable)

Not applicable, no interim analysis will be performed. However, in order to fuel the public debate, injury characteristics and specific treatment characteristics may be analyzed before follow-up is complete.

11. ETHICAL CONSIDERATIONS

11.1 Regulation statement

The study will be conducted according to the principles of the Declaration of Helsinki (64th World Medical Association General Assembly, Fortaleza, Brazil, October 2013). This study has been exempted by the medical research ethics committee (MREC); in Dutch: Medisch Ethische Toetsings Commissie (METC). Following review of the protocol, the MREC concluded that this study is not subject to the Medical Research Involving Human Subjects Act (WMO). They concluded that the study is a medical/scientific research, but no patients are subjected to procedures or are required to follow rules of behavior.

11.2 Recruitment and consent

Eligible persons with fireworks-related injuries (or the parents of pediatric patients) will be informed about the study as soon as possible after hospital presentation. They may receive the information at the ED/ward or at home. The local staff will ask permission to send contact details to the research team. Upon receipt of that permission, a local contact person in the participating hospital will provide details of the patient to the research team. The coordinating investigator or research assistant will send the patient (or parents) the information brochure. The coordinating investigator or a research assistant will visit the patient at the ward/outpatient clinic or at home within two weeks after presentation, in order to further explain the study, answer any questions the patient (or parents) may have, and for signing informed consent. This gives patients up to two weeks to consider their participation. Should patients decline participation only due to the burden of the questionnaires, consent will be asked to only use the information registered in the medical files.

Participation in this study is on a voluntary basis. If persons do not wish to participate, they can do so without specifying why. Deciding not to participate in the study will not affect regular treatment and follow-up care. Participants are allowed to withdraw from the study at any time after they have given their written consent.

Participation in this study will not have any influence on treatment. The General Practitioner (GP) will be informed about the participation of his/her patient.

11.3 Objection by minors or incapacitated subjects (if applicable)

Pediatric patients are involved in this study. Should pediatric patients show any sign of objection, they will not undergo examination of scar tissue quality.

Adult patients will only be enrolled if they are able to provide informed consent themselves. They cannot be incapacitated.

11.4 Benefits and risks assessment,

Management (resuscitation, diagnostics, treatment, after-care, and follow-up visits) of study participants will not differ from those who do not participate. The study does not interfere with diagnostics, treatment, and follow-up at all. As part of this study, patients will be asked to complete questionnaires directly after inclusion (and within two weeks) and after three, six and after 12 months. Physical examination will be performed in patients with burn wounds to assess the scar quality at three, six and 12 months.

All clinical data will be collected during the follow-up visits as well as from the patients' medical files. Therefore, risks associated with participation can be considered negligible.

Participants should not expect any personal benefits from their participation in this study. Their participation may help future patients admitted with fireworks-related injuries. There are no other known additional risks involved in this study, aside from the slight inconvenience of completing the questionnaires. This will take approximately 30 minutes each time, including regular follow-up. The total time investment per patient is thus 90 minutes for three follow-up moments.

Burden

Adult patients and parents of pediatric patients are asked to complete the EQ-5D-3L (adult, youth or youth-proxy version). Depending their trauma patients have to complete injury-specific questionnaires. Patients with burn wounds will be subjected to physical examination regarding scar quality. The burden of completing the questionnaires and the physical examination can be considered as minimal.

Group relatedness

This study is group-related and cannot be conducted without the participation of pediatric patients. Gaining inside into the epidemiology of fireworks-related injuries, treatment, and outcome in pediatric patients can only be studied by enrolling those pediatric patients.

11.5 Compensation for injury

As the study is not subject to the Medical Research Involving Human Subjects Act (WMO), the statutory obligation to provide insurance for subjects participating in medical research (article 7 of the WMO) also does not apply.

11.6 Incentives (if applicable)

There is no incentive for patients to participate in this study. There are no costs involved for study participants. The motivation for the study is a potential benefit to all future patients admitted with fireworks-related injuries, as the results of this study will increase our knowledge on the epidemiology, costs and outcomes of fireworks-related injuries.

12. ADMINISTRATIVE ASPECTS, MONITORING AND PUBLICATION

12.1 Handling and storage of data and documents

Research data will be stored in a database (*e.g.*, OpenClinica Clinical Trial Software for Electronic Data Capture or a similar platform that meets GCP standards and has an audit trail), and will be handled confidentially and anonymously. Research data that can be traced to individual persons can only be viewed by authorized personnel. These persons are the members of the research team, members of the health care inspection, and members of the Medical Research Ethics Committee of the participating hospital. Data review may be necessary in order to ensure the reliability and quality of the research. The handling of personal data is in compliance with the Dutch Data Protection Act (in Dutch: 'Wet Bescherming Persoonsgegevens', Wbp).

Research data will be stored under a code number that relates to individual persons. In order to trace data to an individual subject, a subject identification code list will be made to link the data to the subject. This code consists of the study acronym, three letters for the admitting hospital and three numbers for patient's study ID (*e.g.*, ROCKET-EMC-001 for the first patient at Erasmus MC). Only the code number will be used for study documentation, annual progress reports and research publications. The principal investigator and research assistant are the only persons with the information linking individual persons to study code numbers. This data will be stored during the investigation and will be disposed of 15 years after completion of the study.

12.2 Monitoring and Quality Assurance

Any information on paper collected during this study will be placed in a research folder, which will be filed in locked cabinets in the research offices at Erasmus MC. Any electronic information collected during this study will be saved in a password-protected area. Only the study staff will have access to these data.

Patients will be asked for permission to store the informed consent, contact details, and CRFs at Erasmus MC, as the coordinating investigator or research assistant will contact patients during the study and will collect their follow-up data.

One of the project leaders or an independent person will monitor quality of the data by checking entry of data into the database. Data entry of injury details for a random selection of 25% of patients will be checked. In addition, data entry of questionnaires will be checked for 10% of all enrolled patients.

The questionnaires that patients and the coordinating investigator or research assistant complete during follow-up will be considered as source documents. With

respect to the clinical data such as surgical details, adverse events and interventions, the (electronic) patients files and case report forms will serve as source data.

12.3 Amendments

Amendments are changes made to the research after a favorable opinion by the accredited MREC has been given. All amendments will be notified to the MREC that gave a favorable opinion.

Non-substantial amendments will not be notified to the accredited MREC, but will be recorded and filed by the sponsor. Examples of non-substantial amendments are typing errors and administrative changes like changes in names, telephone numbers and other contact details of involved persons mentioned in the submitted study documentation.

12.4 Annual progress report

Not applicable, as this study will not be subject to the Medical Research Involving Human Subjects Act (WMO).

12.5 Temporary halt and (prematurely) end of study report

No applicable, as this is not an intervention study.

Within one year after the end of the study (*i.e.*, completing data collection), the investigator will submit a final study report with the results of the study, including any publications/abstracts of the study, to the accredited MREC.

12.6 Public disclosure and publication policy

Research data will be reported following the STrengthening the Reporting of OBservational studies in Epidemiology (STROBE) guidelines.

Research data can be presented or publicized in agreement with the principal investigator and project leaders only. Radiographs, CT images, and digital photographs will not be used for publication or teaching purposes without given consent by the patient. No research data that can be traced to individual persons will be presented or published.

The primary publication will be made by the principle investigator. Each participating hospital will provide one co-author for a collaborator group authorship (or full authorship if the journal allows it). This way, hospitals will be acknowledged for their participation.

This study will be registered at The Netherlands National Trial Register (NTR) (in Dutch; Nederlands Trial Register; www.trialregister.nl).

13. STRUCTURED RISK ANALYSIS

Not applicable, as this study does not involve an investigational or non-investigational product (see chapter 6 and 7).

13.1 Potential issues of concern

Not applicable, as this study does not involve an investigational or non-investigational product.

a. Level of knowledge about mechanism of action

Not applicable.

b. Previous exposure of human beings with the test product(s) and/or products with a similar biological mechanism

Not applicable.

c. Can the primary or secondary mechanism be induced in animals and/or in *ex-vivo* human cell material?

Not applicable.

d. Selectivity of the mechanism to target tissue in animals and/or human beings

Not applicable.

e. Analysis of potential effect

Not applicable.

f. Pharmacokinetic considerations

Not applicable.

g. Study population

Not applicable.

h. Interaction with other products

Not applicable.

i. Predictability of effect

Not applicable.

j. Can effects be managed?

Not applicable.

13.2 Synthesis

Not applicable, as this study does not involve an investigational or non-investigational product.

14. REFERENCES

1. de Faber JT. [Fireworks injuries treated by Dutch ophthalmologists New Year 2008/'09] Vuurwerkletsels behandeld door oogartsen: jaarwisseling 2008/'09. Ned Tijdschr Geneeskd. 2009;153:A507.
2. Nijman SV, H. Ongevallen met vuurwerk 2016-2017. Veiligheidnl. 2017.
3. Sandvall BK, Jacobson L, Miller EA, Dodge RE, 3rd, Alex Quistberg D, Rowhani-Rahbar A, et al. Fireworks type, injury pattern, and permanent impairment following severe fireworks-related injuries. Am J Emerg Med. 2017.
4. Tu Y. Fireworks-Related Deaths and Emergency Department-Treated Injuries During 2015. 2016.
5. Canner JK, Haider AH, Selvarajah S, Hui X, Wang H, Efron DT, et al. US emergency department visits for fireworks injuries, 2006-2010. J Surg Res. 2014;190(1):305-11.
6. Chaparro-Narvaez P, Cotes-Cantillo K, Castaneda-Orjuela C, De la Hoz-Restrepo F. Injuries due to fireworks use: A surveillance data analysis in Colombia, 2008-2013. Burns. 2017;43(1):149-56.
7. Moore JX, McGwin G, Jr., Griffin RL. The epidemiology of firework-related injuries in the United States: 2000-2010. Injury. 2014;45(11):1704-9.
8. Valkenberg HN, S. Vuurwerkongevallen 2015-2016. Veiligheidnl. 2016.
9. van der Zee C, Smeulders M, van de Kar A. [Hand injuries caused by fireworks and treated by plastic surgeons] Vuurwerkhandletsel behandeld door plastisch chirurgen. Ned Tijdschr Geneeskd. 2014;158:A8381.
10. Centers for Disease C, Prevention. Injuries from fireworks in the United States. MMWR Morb Mortal Wkly Rep. 2000;49(24):545-6.
11. Roca JB, de los Reyes VC, Racelis S, Deveraturda I, Sucaldito MN, Tayag E, et al. Fireworks-related injury surveillance in the Philippines: trends in 2010-2014. Western Pac Surveill Response J. 2015;6(4):1-6.
12. Van der Sterre GWVdG, E.S. National Eye Institute Visual Functioning Questionnaire - 25; Nederlandse Consensus Vertaling. 2001.
13. Lamers LM, Stalmeier PF, McDonnell J, Krabbe PF, van Busschbach JJ. [Measuring the quality of life in economic evaluations: the Dutch EQ-5D tariff]. Ned Tijdschr Geneeskd. 2005;149(28):1574-8.
14. Beaton DE, Wright JG, Katz JN, Upper Extremity Collaborative G. Development of the QuickDASH: comparison of three item-reduction approaches. J Bone Joint Surg Am. 2005;87(5):1038-46.

15. Irwin MS, Gilbert SE, Terenghi G, Smith RW, Green CJ. Cold intolerance following peripheral nerve injury. Natural history and factors predicting severity of symptoms. J Hand Surg Br. 1997;22(3):308-16.
16. Ruijs AC, Jaquet JB, Daanen HA, Hovius SE. Cold intolerance of the hand measured by the CISS questionnaire in a normative study population. J Hand Surg Br. 2006;31(5):533-6.
17. Binkley JM, Stratford PW, Lott SA, Riddle DL. The Lower Extremity Functional Scale (LEFS): scale development, measurement properties, and clinical application. North American Orthopaedic Rehabilitation Research Network. Phys Ther. 1999;79(4):371-83.
18. Hoogeboom TJ, de Bie RA, den Broeder AA, van den Ende CH. The Dutch Lower Extremity Functional Scale was highly reliable, valid and responsive in individuals with hip/knee osteoarthritis: a validation study. BMC Musculoskelet Disord. 2012;13:117.
19. van de Kar AL, Corion LU, Smeulders MJ, Draaijers LJ, van der Horst CM, van Zuijlen PP. Reliable and feasible evaluation of linear scars by the Patient and Observer Scar Assessment Scale. Plast Reconstr Surg. 2005;116(2):514-22.
